# A transcription factor-mediated regulatory network controls fungal pathogen colonization of insect body cavities

**DOI:** 10.1128/mbio.03504-23

**Published:** 2024-05-15

**Authors:** Juan Deng, Shuaishuai Huang, Yanze Kan, Yue Song, Xin Zhao, Ning Li, Xuewen Yao, Zhibing Luo, Yongjun Zhang

**Affiliations:** 1 Key Laboratory of Agricultural Biosafety and Green Production of Upper Yangtze River (Ministry of Education), College of Plant Protection, Southwest University, Chongqing, China; 2 Key Laboratory of Entomology and Pest Control Engineering, Academy of Agricultural Sciences, Southwest University, Chongqing, China; 3 Beibei Culture Collection of Chongqing Agricultural Microbiology, Southwest University, Chongqing, China; 4 Ministry of Education Key Laboratory of Biodiversity and Eco-Environmental Protection of the Qinghai-Tibetan Plateau, School of Ecology and Environment, Tibet University, Tibet, China; Hebrew University of Jerusalem, Rehovot, Israel

**Keywords:** insect fungal pathogen, hemocoel colonization, transcription factor, regulatory network

## Abstract

Successful host tissue colonization is crucial for fungal pathogens to cause mycosis and complete the infection cycle, in which fungal cells undergo a series of morphological transition-included cellular events to combat with hosts. However, many transcription factors (TFs) and their mediated networks regulating fungal pathogen colonization of host tissue are not well characterized. Here, a TF (BbHCR1)-mediated regulatory network was identified in an insect pathogenic fungus, *Beauveria bassiana*, that controlled insect hemocoel colonization. *BbHCR1* was highly expressed in fungal cells after reaching insect hemocoel and controlled the yeast (*in vivo* blastospores)-to-hyphal morphological switch, evasion of immune defense response, and fungal virulence. Comparative analysis of RNA sequencing and chromatin immunoprecipitation sequencing identified a core set of BbHCR1 target genes during hemocoel colonization, in which *abaA* and *brlA* were targeted to limit the rapid switch from blastospores to hyphae and fungal virulence. Two targets encoding hypothetical proteins, HP1 and HP2, were activated and repressed by BbHCR1, respectively, which acted as a virulence factor and repressor, respectively, suggesting that BbHCR1 activated virulence factors but repressed virulence repressors during the colonization of insect hemocoel. BbHCR1 tuned the expression of two dominant hemocoel colonization-involved metabolite biosynthetic gene clusters, which linked its regulatory role in evasion of immune response. Those functions of BbHCR1 were found to be collaboratively regulated by Fus3- and Hog1-MAP kinases via phosphorylation. These findings have drawn a regulatory network in which Fus3- and Hog1-MAP kinases phosphorylate BbHCR1, which in turn controls the colonization of insect body cavities by regulating fungal morphological transition and virulence-implicated genes.

**IMPORTANCE:**

Fungal pathogens adopt a series of tactics for successful colonization in host tissues, which include morphological transition and the generation of toxic and immunosuppressive molecules. However, many transcription factors (TFs) and their linked pathways that regulate tissue colonization are not well characterized. Here, we identified a TF (BbHCR1)-mediated regulatory network that controls the insect fungal pathogen, *Beauveria bassiana*, colonization of insect hemocoel. During these processes, BbHCR1 targeted the fungal central development pathway for the control of yeast (blastospores)-to-hyphae morphological transition, activated virulence factors, repressed virulence repressors, and tuned the expression of two dominant hemocoel colonization-involved immunosuppressive and immunostimulatory metabolite biosynthetic gene clusters. The BbHCR1 regulatory function was governed by Fus3- and Hog1-MAP kinases. These findings led to a new regulatory network composed of Fus3- and Hog1-MAP kinases and BbHCR1 that control insect body cavity colonization by regulating fungal morphological transition and virulence-implicated genes.

## INTRODUCTION

Successful host tissue colonization is crucial for pathogenic fungi-included microbial parasites to complete the infection cycle. Fungal pathogens infect their hosts via penetration of the host body wall, which is involved in a series of body wall-degrading enzymes and mechanical pressure generated by the formed infection structures, such as appressoria and penetration pegs. Once reaching host tissues, fungal pathogens form different morphological cells, e.g., yeast cells, for tissue colonization. During this process, fungal pathogens have evolved diverse tactics to evade, inhibit, detoxify, and/or eliminate host immune responses (1–8). With the aid of a series of hydrolases, fungal pathogens assimilate host nutrients and lead to host death or tissue necrosis (2, 3, 9, 10). Therefore, besides host body wall penetration, fungal colonization of tissues/host body cavities determines the development of mycosis.

Some conserved or species-specific transcription factors (TFs) have been characterized in fungal pathogens during tissues/host body cavity colonization-involved morphological transition and cellular events. TF Rim101 affects the yeast-hypha morphological transition, which targets cell wall structure-associated genes to control the interaction between *Candida albicans* and oral epithelial cells (11). Ryp1-4 (required for the yeast phase) in *Histoplasma capsulatum* promotes the transition to yeast cells and affects pathogenicity (12). Ryp1 in *Coccidioides posadasii* regulates morphology-related genes, e.g., key virulence factor SOWgp (spherule outer wall glycoprotein), and controls the mature spherule formation and full virulence (13). In *Aspergillus fumigatus*, AcuK and AcuM form a complex to stimulate unique transcriptional regulatory pathways and regulate gluconeogenesis and virulence during *in vivo* infection (14). Another two highly expressed TFs, rlmA and sltA, during *A. fumigatus* growth *in vivo* contribute to fungal pathogenicity. The former is required for fungal burden in the lung, and the latter regulates the expression of multiple secondary metabolite gene clusters and ribotoxin aspf1 (15). When entering the insect hemocoel after cuticle penetration, the insect fungal pathogen *Metarhizium robertsii* upregulates the epigenetically controlled factor COH1 to suppress the expression of cuticle-degrading genes by blocking the specific regulator COH2 and upregulates the genes for hemocoel colonization (16). These results suggest multiple regulatory roles of TF-mediated networks in tissue colonization-associated cellular events, although details of many TFs linked with signaling pathways remain limited. However, compared to over 37 different TF families and an average of ~360 different TFs encoded in Ascomycete genomes, which can range from as few as 113 in *Schizosaccharomyces japonicus* to 1,035 in *Nectria haematococca* (17), the contribution of other TFs to fungal colonization of host tissues/body cavities remains unknown.

*Beauveria bassiana* is a broad-spectrum important entomopathogenic fungus that has been used for the biocontrol of a wide range of arthropod pests as a microbial biological agent (18, 19). Like many fungal pathogens of plants/mammals, *B. bassiana* and other insect fungal pathogens infect their hosts via direct penetration of the cuticle, which is jointly acted upon by enzymatic activities, mechanical pressure, and the secretion of (toxic) secondary metabolites (20, 21). Once reaching the insect hemocoel, the infective hyphae differentiate to *in vivo* blastospores (termed hyphal bodies) to colonize the hemocoel, where fungal cells adopt strategies, i.e., remodeling and camouflaging cell walls and producing effectors, insecticidal and immunosuppressive metabolites to evade host innate immune recognition, and suppress or interfere with the insect immune defense response, which helps fungal cells rapidly propagate via *in vivo* blastospores, eventually developing diseases. After killing the insect, fungal cells switch from *in vivo* blastospores to hyphae to assimilate host nutrition and then penetrate outward from the cadavers for growth and conidiation to disperse (5, 8, 10, 22). Although some signal molecules, TFs, and other molecules involved in fungal morphological transition and evasion of immune responses have been characterized in insect fungal pathogens *Metarhizium* and *Beauveria* species (10, 23), other TFs or their mediated networks that regulate insect hemocoel colonization in those fungal species are not well characterized.

In this study, we characterized a Zn(II)_2_Cys_6_ TF, BbHCR1, in *B. bassiana*, which regulated insect hemocoel colonization by regulating fungal dimorphic transition and development via targeting asexual developmental regulatory factor genes, *brlA* and *abaA*, and virulence-associated genes, as well as indirect control of the biosynthesis of immunosuppressive metabolites, oosporein and beauverolide. The regulatory function of BbHCR1 was collaboratively regulated by Fus3- and Hog1-MAP kinases via phosphorylation. These data illustrated a new TF-mediated regulatory network that controls *B. bassiana* colonization of insect hemocoel and virulence.

## RESULTS

### Identification of TFs involved in *B. bassiana* colonization of insect body cavity

**Fig 1:**
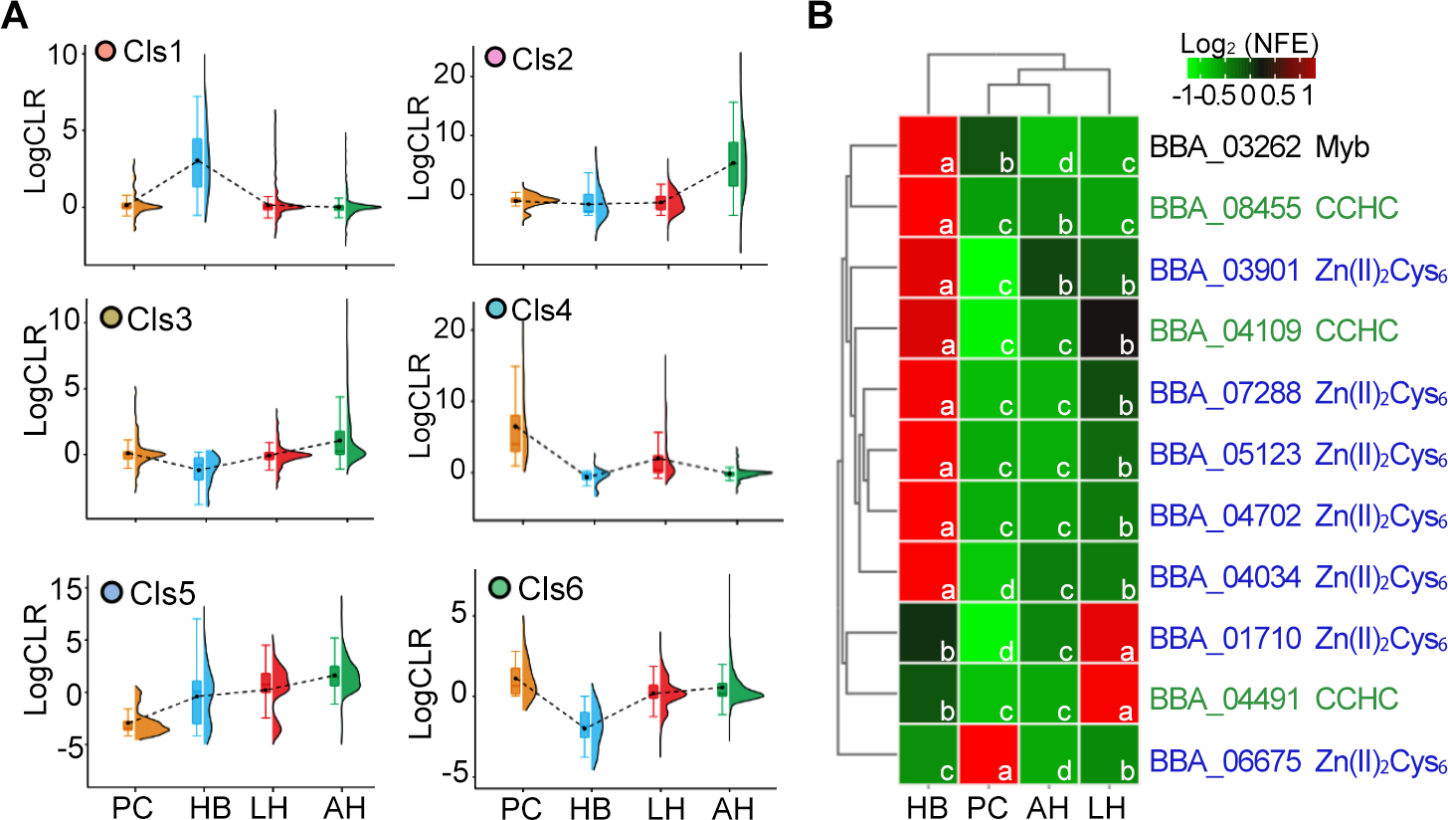
Identification of *Beauveria bassiana* TFs involved in insect hemocoel colonization. (**A**) Clusters of global co-expression TF genes with similar expression patterns. Mean expression values of genes in each cluster were linked by a black dashed line. PC, HB, LH, and AH indicate fungal cells from the penetration of (insect) cuticle, hyphal bodies (*in vivo* blastospores), liquid hyphae, and aerial hyphae, respectively. (**B**) The RT-qPCR analysis of the expression profile of 11 TF genes (along with the accession number and type of TF) that are highly expressed in HB from Cls1 in the Bb0062 strain using *18S rRNA* as a reference gene. Different letters (a–d) indicate statistically significant differences (*P* < 0.01 in the LSD test).

The gene expression data of 559 *B. bassiana* TFs were retrieved from the whole genome expression database that was constructed from 76 deep-sequenced RNA-seq samples covering fungal cells derived from cells in penetration of (insect) cuticle (PC), *in vivo* blastospores (hyphal bodies [HB]), liquid hyphae (LH), and aerial hyphae (AH) of *B. bassiana* (24). Co-expression analysis revealed that those TF genes with similar expression patterns were enriched into six clusters (Cls1–Cls6) (Fig. S1). Cls1, Cls2, and Cls4 represented TF genes that were highly expressed in HB, AH, and PC, respectively (Fig. 1A). TF genes in Cls3 were slightly downregulated in HB but slightly upregulated in AH, while those TF genes in Cls5 and Cls6 were downregulated in PC and HB, respectively (Fig. 1A). Although the transcripts of Cls1 TF genes were detected in PC, HB, LH, and AH, the relative expression levels were dramatically increased in HB (average LogCLR = 3.01 ± 0.48) as compared with the other three types of cells (PC 0.10 ± 0.07, LH 0.09 ± 0.50, and AH −0.01 ± 0.23). The Cls1 harbored 11 TFs, including 7 Zn(II)_2_Cys_6_, 3 CCHC-type zinc finger proteins, and 1 Myb TF. Expression patterns of those 11 TF genes were verified by RT-qPCR in *B. bassiana* Bb0062 strain. The results indicated that eight genes were highly active in HB, while other two and one genes were highly expressed in LH and PC, respectively (Fig. 1B). These data suggested that those eight TFs might regulate *B. bassiana* colonization of insect hemocoel.

**Fig 2:**
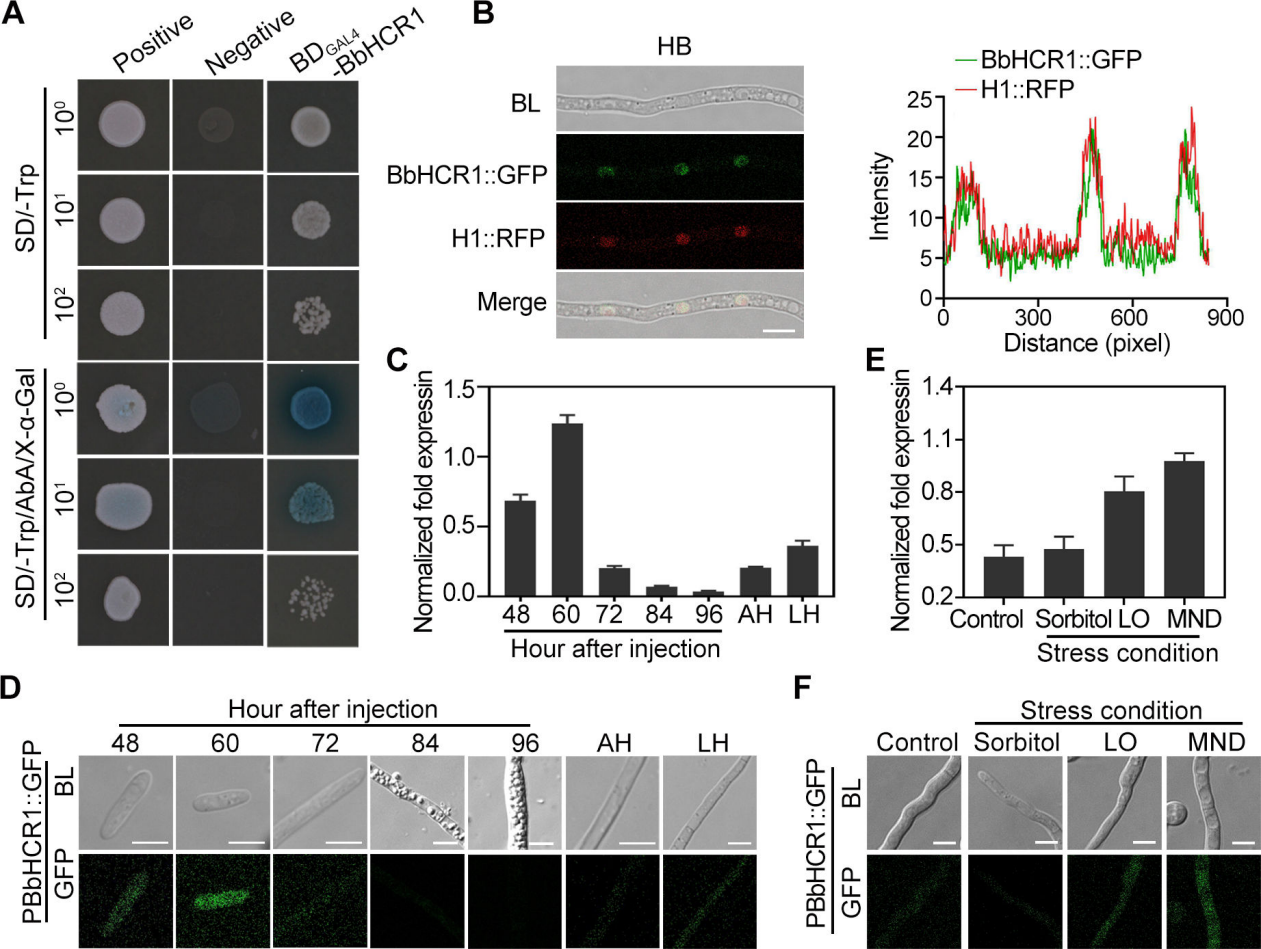
Transcription factor characteristics and expression profiles of BbHCR1. (**A**) Transcriptional activation assays in yeast. Yeast strain Y2HGold expressing the GAL4 activation and the GAL4 DNA-binding domains (positive control), the blank vector with the GAL4 DNA-binding domain (negative control), or the GAL4 DNA binding and BbHCR1 (BD_GAL4_-BbHCR1) were cultured on the medium SD/-Trp and SD/-Trp with 0.35 µg/mL AbA (Aureobasidin A) and X-α-Gal at 30°C for 3 days. (**B**) Subcellular localization of BbHCR1 in *B. bassiana*. Distribution of GFP fluorescence of BbHCR1::GFP hyphal bodies. Nucleus was labeled with RFP by fusion RFP at the C-terminal of histone (H1::RFP). (**C and E**) RT-qPCR analysis of *BbHCR1* expression patterns in fungal cells proliferated in insect hemocoel after the injection of conidia at indicated hours in aerial hyphae and liquid hyphae (**C**) and under stressed conditions (**E**). Fungal cells were cultured in CZB (control) and CZB containing 1.0 M sorbitol, 75 µM menadione (MND), or 1 mM CoCl_2_ for 6 h. (**D and F**) GFP fluorescence of PBbHCR1::GFP strain in panels **C and E**, in which *GFP* was driven by *BbHCR1* promoter. Scale bar = 5 µm.

One of those eight TFs with the tag code BBA_04034, designed as BbHCR1 (*B. bassiana* hemocoel colonization-associated regulator 1), consists of 928 amino acids containing two putative GAL4-like DNA-binding domains at residues 66–108 and 127–171 and a fungal-specific transcription factor domain at residues 332–757, with one predicted nuclear localization signal, PFRRGRS, at residues 798–804 (25). BLASTp analysis revealed that the homologs of BbHCR1 are commonly present in fungal species from yeast to filamentous fungi, whereas phylogenetic separation could be seen with hosts, i.e., insects and fungi (e.g., the hyperparasite fungus *Trichoderma atroviride*), plants, and mammals, despite the yeast *Lipomyces starkeyi* forming a separate branch (Fig. S2). Transcriptional activation assays revealed that yeast cells expressing *BbHCR1* cDNA grew as well on an auxotrophic medium as the positive control yeast cells expressed a GAL4 activation domain with a blue colony (Fig. 2A). GFP fluorescence signals of BbHCR1::GFP cells (HB) were overlapped with a red fluorescent protein (RFP)-tagged H1 (histone), suggesting localization in the nucleus (Fig. 2B). These results indicated that BbHCR1 was a typical TF.

RT-qPCR analysis revealed that expression of *BbHCR1* was significantly elevated in hemolymph-derived cells, HB, at 48–60 h after the injection of conidia, but dramatically decreased after 72 h of inoculation when fungal cells were switched from HB to hyphae. These results were consistent with the GFP fluorescence observation of PBbHCR1::GFP cells, in which the GFP gene was controlled by the BbHCR1 promoter (Fig. 2C and D), suggesting involvement in the early colonization of insect hemocoel. Moreover, *BbHCR1* expression was slightly increased in LH and induced by low oxygen (LO) and oxidative [menadione (MND)] stresses (Fig. 2E and F).

### BbHCR1 controls fungal virulence

**Fig 3:**
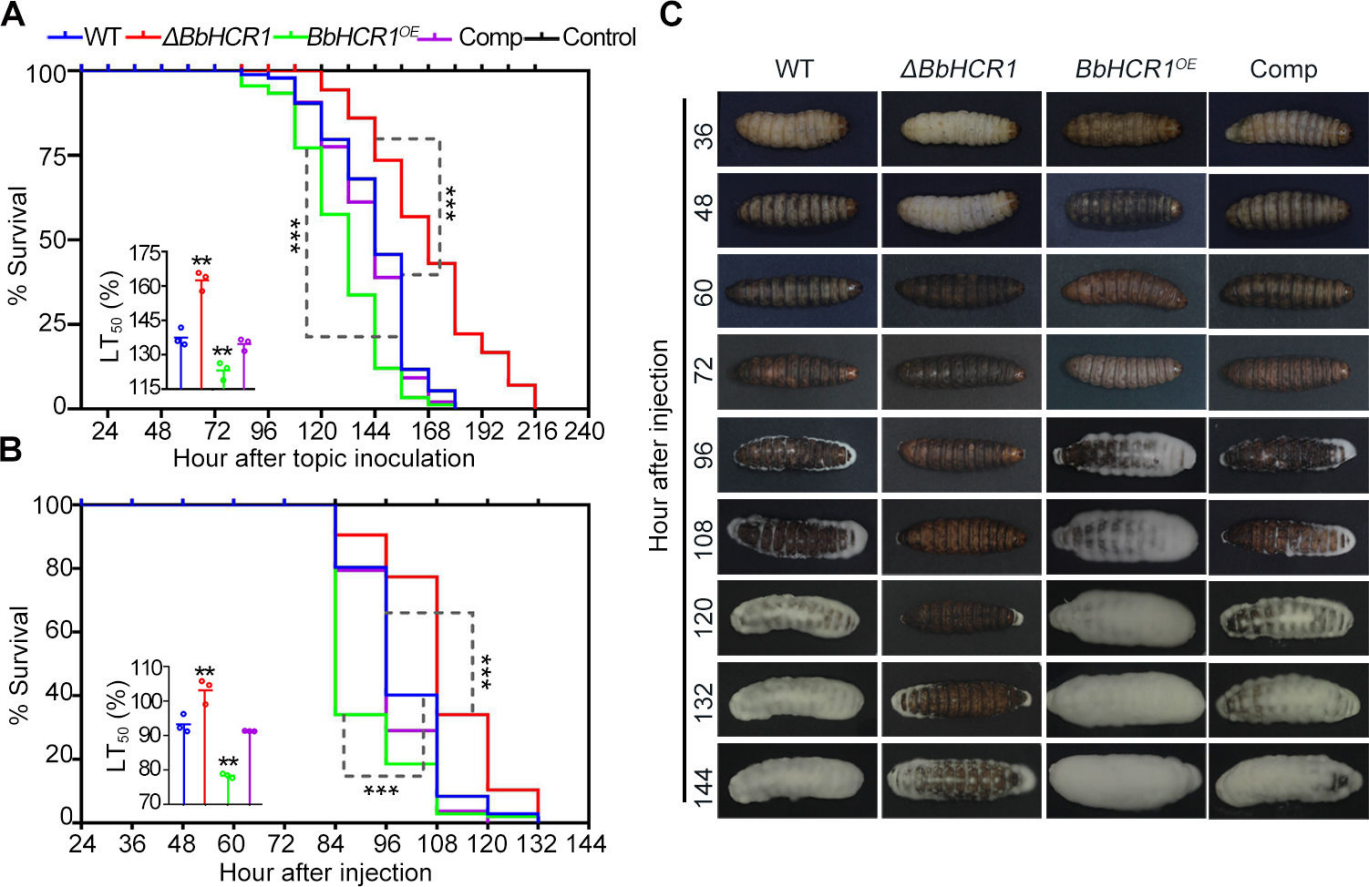
Insect bioassays. (**A and B**) Insect survival and calculated LT_50_ values using *G. mellonella* larvae following the topical application (1 mL of 10^7^ conidia/mL) and intrahemocoel injection of conidia (2 µL of 10^5^ conidia/mL). Control insects were treated with 0.05% Tween-80. *P < 0.05; **P < 0.01; and ***P < 0.001. (**C**) Symptom of *G. mellonella* larvae after hemocoel injection at the indicated time.

To reveal the role of BbHCR1 in *B. bassiana* colonization of insect hemocoel, the gene disruption (Δ*BbHCR1*), reverse complementation (Δ*BbHCR1::BbHCR1*, Comp), and overexpression (*BbHCR1^OE^*) strains were generated as detailed in Materials and Methods and Fig. S3. Disruption of *BbHCR1* did not affect colony growth on agar plates but with increased aerial hyphal development, while reduced conidiation on rich-nutrient 1/4 SDAY cultures (49.5%–56.4%, *P* < 0.01). Whereas overexpression strain displayed slightly reduced growth on agar plates with increased conidia production on 1/4 SDAY (28%–62%, *P* < 0.01) and germination with reduced GT_50_ (the mean 50% conidial germination time) by 20.8% (*P* < 0.01) (Fig. S4). Contribution of BbHCR1 to fungal virulence was assayed using last-instar *Galleria mellonella* larvae via topical cuticle infection and intrahemocoel injection (cuticle-bypassing infection). In both assays, decreased and increased virulence was examined in Δ*BbHCR1* and *BbHCR1^OE^* strains, respectively. The topical bioassay (spraying 1 mL of 10^7^ cells/mL conidia suspension) resulted in the median lethal time to kill 50% of hosts (LT_50_) for the Δ*BbHCR1* = 162.5 ± 3.4 h, which was 1.2-fold higher than wild-type (WT) strain (137.5 ± 3.2 h) (decreased virulence, *P* < 0.01). However, LT_50_ values were decreased to 123.2 ± 3.1 h for the *BbHCR1^OE^* strain (increased virulence, *P* < 0.01) (Fig. 3A). Similar results were examined in bioassays via intrahemocoel injection (2 µL of 10^5^ conidia/mL per larvae) (Fig. 3B). No obvious difference in cuticle penetration was assayed *in vitro* using cicada (*Graptopsaltria nigrofuscata*) hind wings to mimic the insect cuticle (26) (Fig. S5), suggesting that decreased/increased virulence of Δ*BbHCR1*/*BbHCR1*^*OE*^ strains was due to their different abilities to colonize the hemocoel. Unless otherwise noted, in all phenotypes examined, the Comp strain was identical to the WT.

It was noticed that the *BbHCR1^OE^*-infected larvae appeared to have a melanization reaction at 36 h post-injection of conidia and turned black at 48 h, all of which were 12 h ahead of WT. More *BbHCR1^OE^* hyphae penetrated outward from the cadaver for growth 96 h post-inoculation (hpi), whereas little penetrated hyphae of control strains (WT and Comp) were seen on the larvae surface. However, the melanization reaction of the Δ*BbHCR1*-inoculated larvae was delayed by 24 h at least as compared to the control strains, and little hyphae penetrated outward from the cadaver mouthparts and excretory channels were seen until 120 hpi. At 144 hpi, hyphae of the Δ*BbHCR1* grown on the cadaver surface were obviously less than control strains, but the *BbHCR1^OE^* growth was more than control strains (Fig. 3C). These data were consistent with their changes in virulence.

### BbHCR1 is involved in insect hemocoel colonization

**Fig 4:**
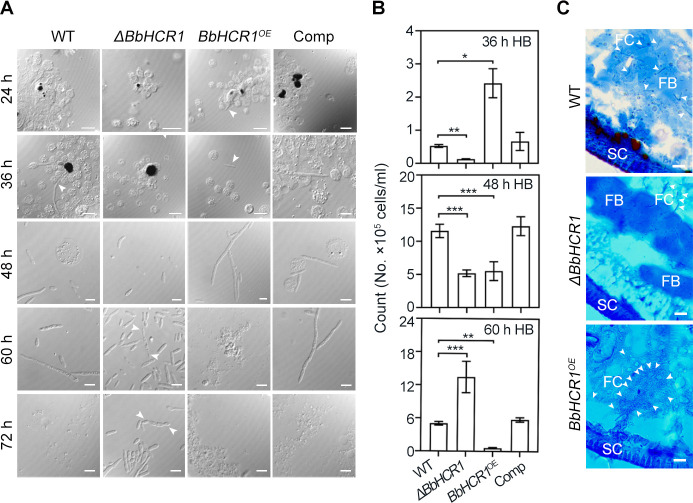
Fungal development and insect immune responses in hemocoel. (**A**) Microscopic images of fungal development and insect immune responses at the indicated time after injection. White arrows indicate fungal cells. Scale bar = 5 µm. (**B**) Quantification of the hyphal bodies from hemocoel at 36, 48, and 60 h after infection using qPCR analysis by amplification of the *18S rRNA* sequence as detailed in Materials and Methods. *P < 0.05; **P < 0.01; and ***P < 0.001. (**C**) Frozen section of larvae at 60 h after the injection of conidia. Sample stained by lactic acid phenol cotton blue solution. SC, insect cuticle. FB, fat body. White arrows indicate fungal cells (FC). Scale bar = 20 µm.

To probe the fungal development in the insect hemocoel, *G. mellonella* larvae were bled after the injection of conidia for microscopy examination. *BbHCR1^OE^* germinated conidia (the germ tubes) more easily escaped hemocyte encapsulation at 24 hpi and freely grew as compared to control strains, which evaded the hemocyte encapsulation after 36 h of inoculation. Although few fungal cells were seen to evade the encapsulation at 36 h after the inoculation of Δ*BbHCR1* conidia, some cells grew freely at 48 hpi and developed numerous *in vivo* blastospores from 60 to 72 hpi (Fig. 4A). However, compared to control strains that formed stick-shaped HB (*in vivo* blastospores) from 48 to 60 hpi, *BbHCR1^OE^*-germinated conidia formed HB at 36 hpi and rapidly developed to hyphae at 48 hpi, with hardly any fungal cells seen in the hemolymph at 60 hpi (Fig. 4A). Changes in free-floating fungal cells in the hemolymph from 36 to 60 hpi were verified by qPCR analysis by amplification of the *18S rRNA* sequence (5) (Fig. 4B). The similar morphological switches were also seen *in vitro* cultures (in 1/4 SDY broth) (Fig. S6A). The examination of the tissue section revealed that more fungal hyphae of *BbHCR1^OE^* were distributed in larval tissues, e.g., fat bodies and subcutaneous tissues, than control strains at 60 hpi (Fig. 4C). These results suggested the role of BbHCR1 in regulating the morphological switch from *in vivo* blastospores to hyphae during hemocoel colonization. Moreover, reduced/increased growth of Δ*BbHCR1/BbHCR1^OE^* cells was also seen in broth with oxidative (75 µM MND) or/and low oxygen (1 mM CoCl_2_, a hypoxia-mimicking agent) agents (Fig. S6), which mimicked stress niches in fungal infected insect hemocoel (27, 28), suggesting linkage of BbHCR1-mediated fungal development to adaptation to infected insect hemocoel niches.

### BbHCR1 targets fungal development and secondary metabolite synthesis genes at the early colonization of insect hemocoel

To reveal the underlying mechanism of BbHCR1 regulating the morphological switch from blastospores (HB) to hyphae and evasion of immune response in the early colonization of insect hemocoel, RNA sequencing (RNA-seq) and chromatin immunoprecipitation (ChIP) sequencing (ChIP-seq) were performed to identify the BbHCR1 target genes. Compared to RNA-seq from normal cultures (1/4 SDY for 3 days), 197 and 210 genes were specifically upregulated and downregulated in the hemolymph-derived Δ*BbHCR1* cells (HB, 48 h after injection of conidia), respectively, while 62 and 21 genes were specifically upregulated and downregulated in *in vivo BbHCR1^OE^* cells as compared to those of WT, respectively (Fig. S7B). Among those differently expressed genes (DEGs) in *in vivo* cells, 28 DEGs displayed opposite expression patterns in the Δ*BbHCR1* and *BbHCR1^OE^* cells, including 11 and 17 DEGs upregulated and downregulated in the Δ*BbHCR1* cells but downregulated and upregulated in the *BbHCR1^OE^* cells, respectively (Fig. S8A). Those DEGs were enriched in nutrient utilization, secondary metabolism, fungal development, and other functions. Those nutrient utilization-involved genes encode hydrolases, such as proteases, peptidases, and glucanases, all of which were significantly upregulated in Δ*BbHCR1* but downregulated in *BbHCR1^OE^* (Fig. S8B). Whereas those secondary metabolism genes were involved in the biosynthesis of oosporein (encoding Ops1, Ops4, Ops6, and Ops7) (29), beauverolide (encoding besA and besB) (30), and other metabolites, which were significantly downregulated in Δ*BbHCR1* but upregulated in *BbHCR1^OE^* cells (Fig. S8B). In those fungal development genes, two cell adhesion-associated genes (BBA_03909 and BBA_09863) and one conidial cell wall protein gene (BBA_07138) were significantly downregulated in Δ*BbHCR1* but upregulated in *BbHCR1^OE^* cells, whereas one cell cycle gene (BBA_02863) and one central development pathway (CDP) regulator abaA gene (BBA_00300) were significantly upregulated in Δ*BbHCR1* but downregulated in *BbHCR1^OE^* cells (Fig. S8B). We further probed the other CDP regulator genes and found that *brlA* was also significantly upregulated Δ*BbHCR1* (log_2_FC = 1.7) but slightly downregulated in *BbHCR1^OE^* cells (log_2_FC = −0.9) (*P* value = 0.07). All those DEGs were associated with cellular events in the early colonization of insect hemocoel.

**Fig 5:**
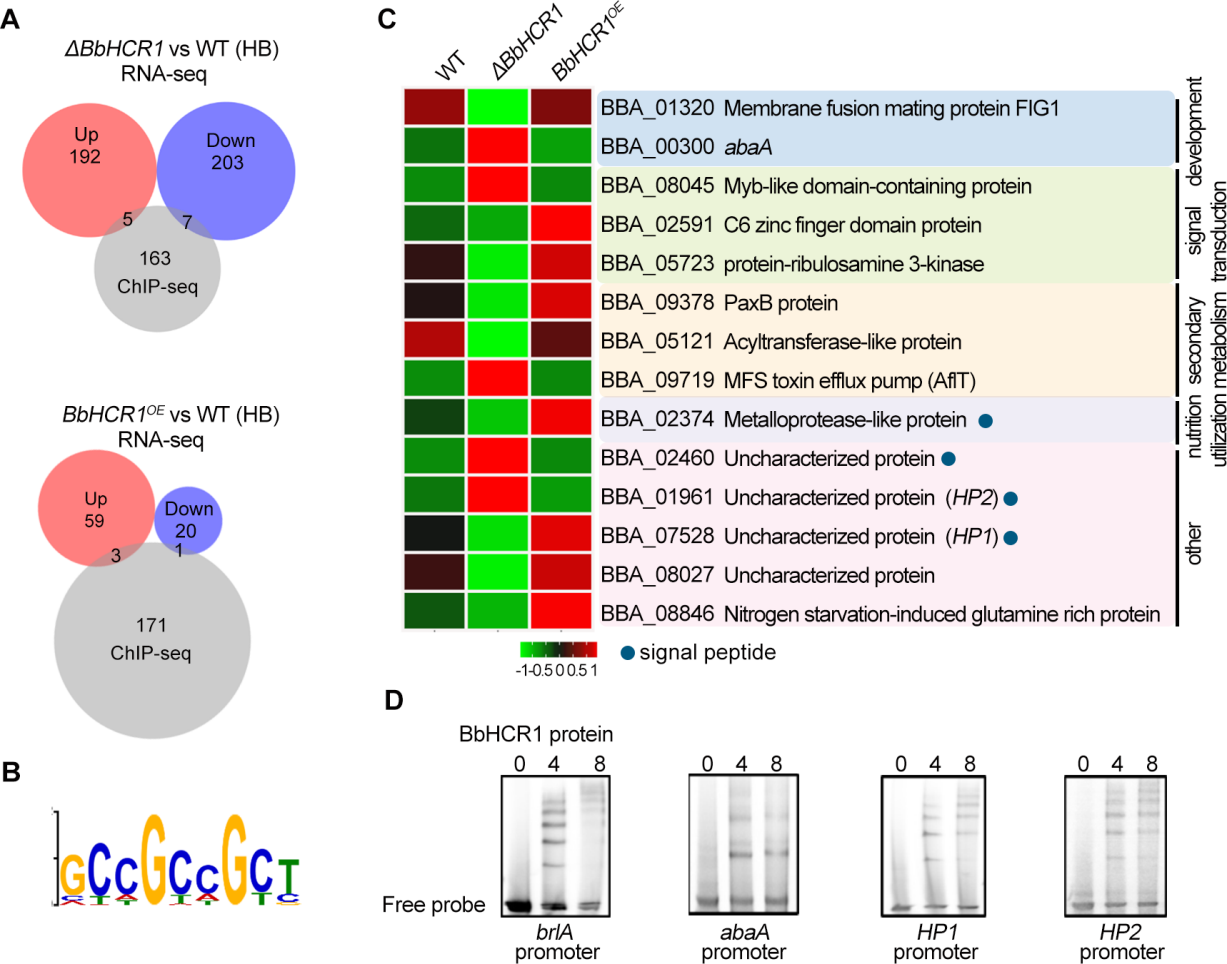
Identification the BbHCR1 target genes by comparative analysis of ChIP-seq and RNA-seq. (**A**) Venn diagram of RNA-seq (|fold change| ≥ 2) and ChIP-seq. (**B**) MEME analysis indicating the likely binding sequence of BbHCR1. (**C**) Annotation of identified BbHCR1 gene targets and their expression patterns. Solid blue circles indicate proteins containing signaling peptides. (**D**) EMSA verification of four identified gene targets of BbHCR1. The promoter fragments containing predicted BbHCR1-binding motifs of four target genes, 472, 322, 328, and 369 bp for *brlA*, *abaA*, *HP1,* and *HP2*, respectively, were amplified and used for EMSA assays with His-tagged DNA-binding domain of BbHCR1 (15.4 kDa), which was expressed in *Escherichia coli* BL21 and purified.

To probe the targets of BbHCR1, ChIP-seq was performed using a Δ*BbHCR1* strain constitutively expressing 13 Myc-tagged BbHCR1 (probed with an anti-Myc antibody) grown in 1/4 SDY. A total of 521 unique ChIP-seq peaks were mapped to 175 different target genes (within 2.0 kb of an ORF start codon) (Fig. 5A). MEME analysis indicated the putative DNA-binding sequence for BbHCR1 to be VYHGYHGYB, with lowest *P* value (1.9e-1) and frequency (75/175) (Fig. 5B). Comparison of the ChIP-seq with the RNA-seq data sets (DEGs specifically expressed in *in vivo* cells) indicated the expression of 14 (8% of the total ChIP set) genes as BbHCR1 dependent, including five/seven upregulated/downregulated genes in Δ*BbHCR1* cells and three/one upregulated/downregulated genes in *BbHCR1^OE^* cells, respectively, in which *abaA* (BBA_00300) and a metalloprotease-like protein gene (BBA_02374) were significantly upregulated/downregulated in Δ*BbHCR1* but downregulated/upregulated in *BbHCR1^OE^* cells, respectively. Two targets, BBA_07528 and BBA_01961, encoding two hypothetical (uncharacterized) proteins containing predicted N-terminal signal sequences, designated as *HP1* and *HP2*, were significantly downregulated (log_2_FC = −3.0)/upregulated (log_2_FC = 2.2) in Δ*BbHCR1* cells but slightly upregulated (log_2_FC = 0.84)/downregulated (log_2_FC = −0.65) in *BbHCR1^OE^* cells, respectively (Fig. 5C). Promoter element scanning revealed that one binding motif for BbHCR1 presented at −3,979 to −3,667 bp upstream of *brlA*-coding region (present in the ChIP-seq data). It is demonstrated that a heterotrimeric transcription factor CCAAT-binding complex regulates asexual reproduction (conidiation) of *A. fumigatus* by binding to the promoter region of *brlA* at −4,421 to −3,175 bp upstream of start site “ATG” and promoter regions of other fungal development genes (*fluG*, *flbD*, and *flbC*) (31), suggesting promoter region of *brlA* is over 4,000 bp. Thus, we considered that *brlA* was also a putative target of BbHCR1. Another target gene involved in fungal development and differentiation (BBA_01320, coding membrane fusion mating protein FIG1) (32) was significantly downregulated in the Δ*BbHCR1* strain. Other targets associated with transcription and signaling transduction, secondary metabolism, and uncharacterized proteins displayed opposite expression patterns between Δ*BbHCR1* and *BbHCR1^OE^* strains (Fig. 5C). Binding of purified BbHCR1 to the promoter regions of four identified putative target genes, including *brlA*, *abaA*, *HP1,* and *HP2,* was verified by electrophoretic mobility shift assay (EMSA) using respective promoter fragments from each gene and the purified protein. We scanned the promoter region of each target and found that three, five, seven, and two BbHCR1-binding motifs were present in probes of *brlA* (472 bp from −3,901 to −3,430 bp), *abaA* (322 bp from −656 to −335 bp), *HP1* (328 bp from −800 to −473 bp), and *HP2* (369 bp from −370 to −2 bp), respectively, which was in line with the several positive signals in EMSA assays (Fig. 5D).

### BbHCR1 targets *brlA* and *abaA* and virulence factor genes to control morphological transition in the insect hemocoel and virulence

**Fig 6:**
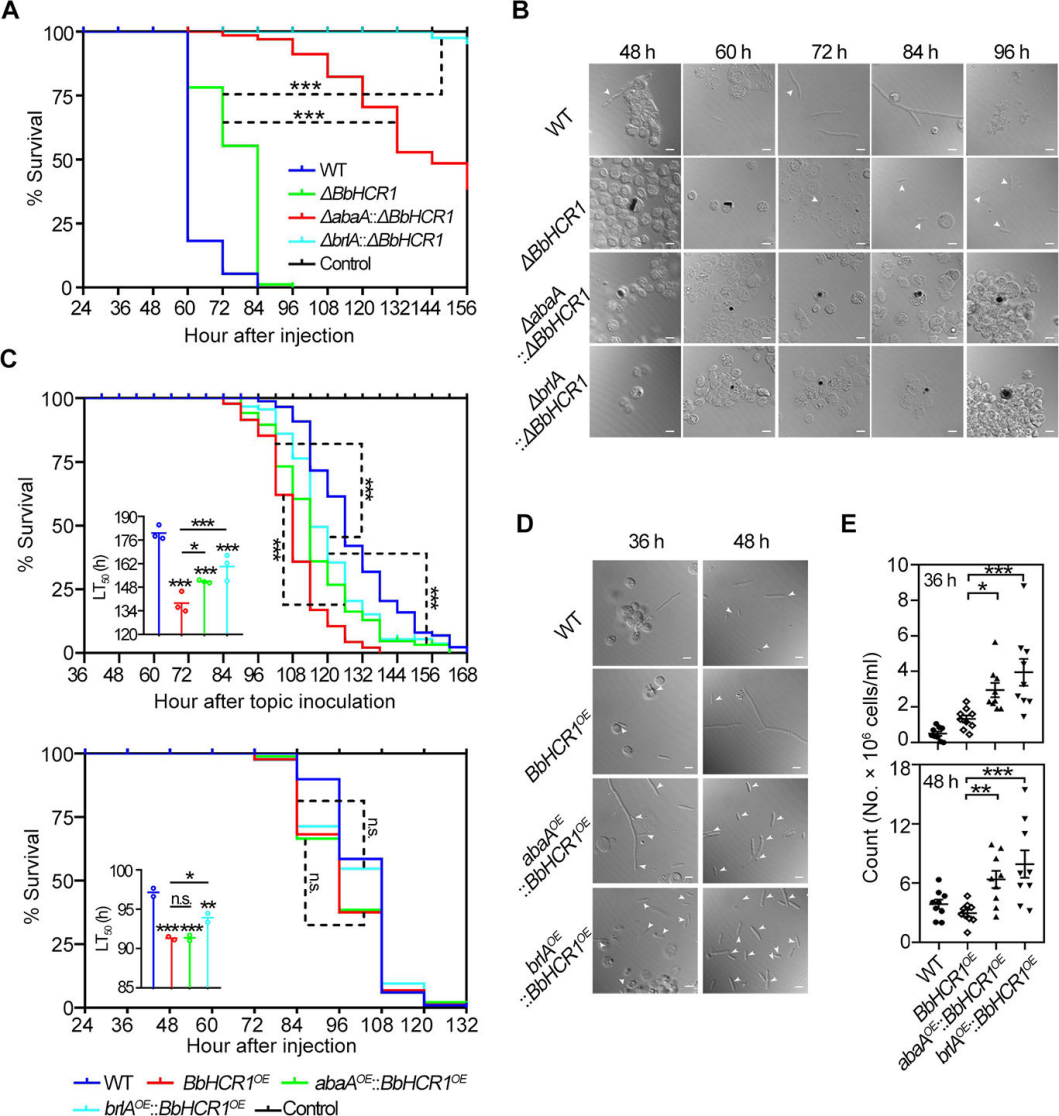
Insect bioassays of WT, Δ*brlA::*Δ*BbHCR1*, Δ*abaA::*Δ*BbHCR1, brlA^OE^::BbHCR1^OE^*, and *abaA^OE^::BbHCR1^OE^* strains. (**A**) Survival of *G. mellonella* larvae following intrahemocoel injection of hyphal fragments (3 µL, 20 mg/mL fresh hyphae) of indicated strain. (**B**) Microscopic images of fungal development and insect immune responses at indicated hours after inoculation. (**C**) Survival of the larvae following the topical application (1 mL of 10^7^ conidia/mL) and intrahemocoel injection (2 µL of 10^5^ conidia/mL) of indicated strains. (**D**) Microscopic images of fungal development following the injection of conidia. White arrows in panels **B** and **D** indicate fungal cells. Scale bar = 5 µm. (**E**) Quantification of blastospores in hemolymph at 36 and 48 h after the injection of indicated strains using a hemocytometer. *P < 0.05; **P < 0.01; and ***P < 0.001.

To reveal linkage of BbHCR1-regulated morphological switch from *in vivo* blastospores to hyphae with its targets, *brlA* and *abaA*, Δ*brlA::*Δ*BbHCR1* or Δ*abaA::*Δ*BbHCR1* mutants, and *brlA^OE^::BbHCR1^OE^* or *abaA^OE^::BbHCR1^OE^* strains were generated by the disruption of *brlA* or *abaA* in Δ*BbHCR1* background and overexpression of *brlA* or *abaA* in *BbHCR1^OE^* background, respectively. Since the resultant Δ*brlA::*Δ*BbHCR1* and Δ*abaA::*Δ*BbHCR1* lost capacities to sporulate either on agar or in broth, their hyphal fragments (20 mg/mL fresh hyphae) were used for insect bioassay by microinjection into the larvae hemocoel. As compared to the Δ*BbHCR1* strain whose virulence was significantly lower than WT, dramatically decreased virulence was detected in the Δ*brlA::*Δ*BbHCR1* and Δ*abaA::*Δ*BbHCR1* strains, in which the former virulence was significantly lower than the latter (Fig. 6A). Unlike the Δ*BbHCR1* hyphal fragments that formed *in vivo* blastospores at 72 hpi, hyphal fragments of Δ*brlA::*Δ*BbHCR1* and Δ*abaA::*Δ*BbHCR1* were encapsulated by hemocytes with a melanization reaction until 96 hpi (Fig. 6B). With respect to *brlA^OE^::BbHCR1^OE^* and *abaA^OE^::BbHCR1^OE^* strains, topical cuticle inoculation and intrahemocoel injection were performed for bioassays. Although the virulence of the two overexpression strains was increased as compared to WT, the *brlA^OE^::BbHCR1^OE^* was less virulent than the *BbHCR1^OE^* strain (*P* < 0.01 and *P* < 0.05 in topical cuticle inoculation and intrahemocoel injection bioassays, respectively), while *abaA^OE^::BbHCR1^OE^* displayed slightly decreased virulence in topical inoculation bioassay (*P* < 0.05) but not in intrahemocoel injection bioassay as compared to the *BbHCR1^OE^* strain (Fig. 6C). However, the morphological switch of *BbHCR1^OE^* cells from *in vivo* blastospores to hyphae was restored to wild type by overexpression of *brlA* or *abaA* in *BbHCR1^OE^* strain (Fig. 6D and E). These results suggested that BbHCR1 targeted *brlA* and *abaA* to control the morphological transition from blastospores to hyphae in the infected insect blood.

To reveal the contributions of BbHCR1 targets to fungal colonization of insect hemocoel and virulence, two secreted protein genes, *HP1* and HP2, which displayed opposite expression patterns in Δ*BbHCR1* and *BbHCR1^OE^* strains during the early hemocoel colonization, with the former being significantly repressed in Δ*BbHCR* cells but slightly upregulated in *BbHCR1^OE^* cells, while the latter being significantly upregulated in the Δ*BbHCR* cells but slightly decreased in *BbHCR1^OE^* cells, were selected as representatives to be characterized. The structures of the two proteins are shown in Fig. S9A. Both *HP1* and *HP2* were highly expressed in WT HB (*in vivo* blastospores, 48 h of inoculation) (Fig. S9B). As expected, disruption of *HP1* (Δ*HP1*) significantly decreased *B. bassiana* virulence, while significantly decreased virulence was examined in the *HP2* overexpression strain (*HP2^OE^*), which resulted in increased LT_50_ values for Δ*HP1/HP2*^*OE*^ strains in both bioassays (Fig. S9C through F). No obvious difference in cuticle penetration was assayed between *HP1* and *HP2* disruption or overexpression strains and WT (Fig. S5). These results suggested that BbHCR1 might activate virulence genes (e.g., *HP1*) but repress virulence-repressor-associated genes (e.g., *HP2)* during insect hemocoel colonization.

### BbHCR1 is phosphorylated by Fus3- and Hog1-MAP kinases

**Fig 7:**
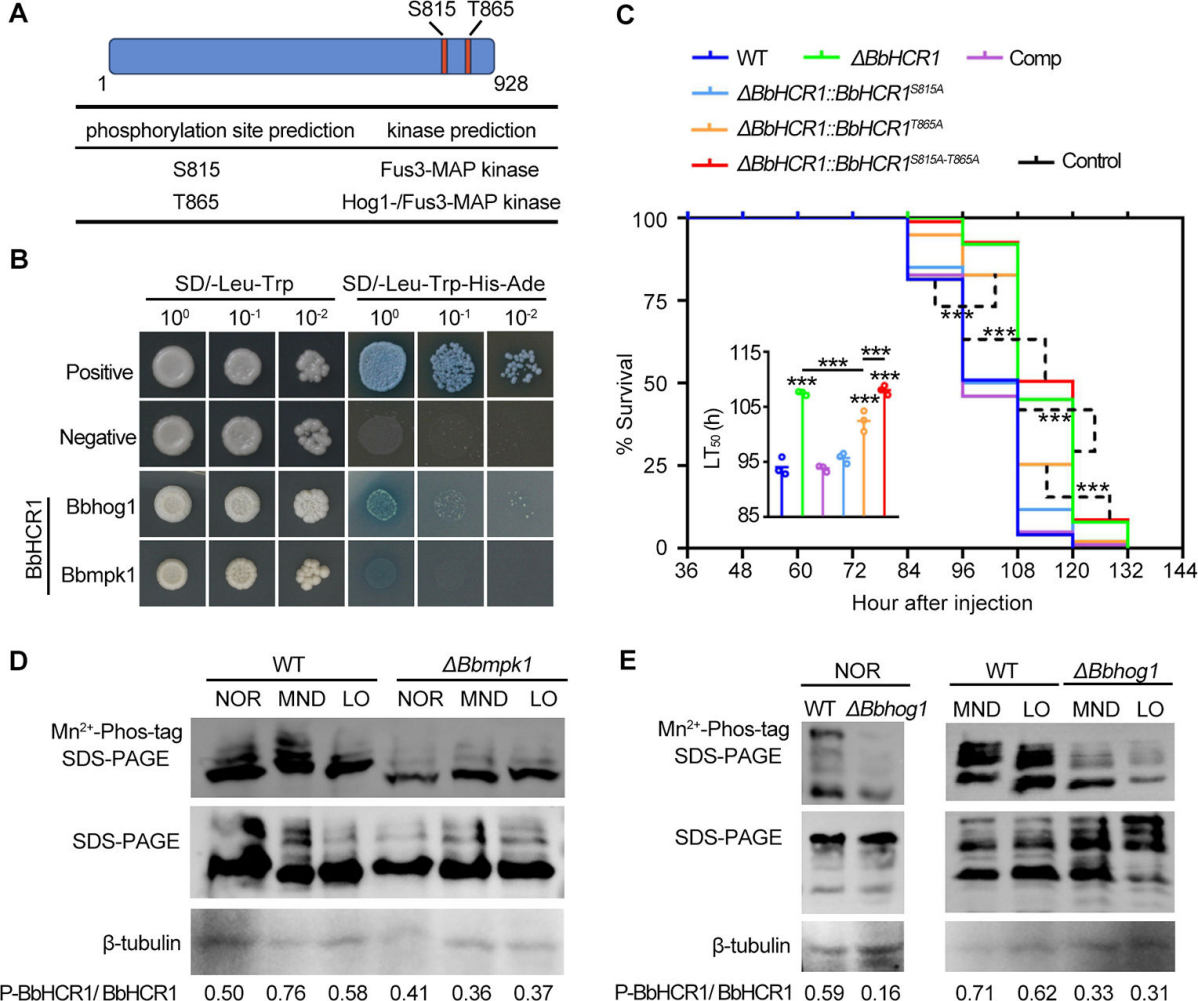
BbHCR1 function in fungal virulence depends on phosphorylation by Fus3-MAP kinase (Bbmpk1) and Hog1-MAP kinase (Bbhog1). (**A**) Predicted phosphorylation sites of BbHCR1 for the possible kinases. (**B**) Yeast two-hybrid tests of the interaction of BbHCR1 and the predicted kinases. (**C**) Survival of *G. mellonella* larvae and the calculated LT_50_ values following intrahemocoel injection (2 µL of 10^5^ conidia/mL) of the indicated fungal conidia. (**D and E**) Assays for the phosphorylation of BbHCR1 in WT, Δ*Bbmpk1,* and Δ*Bbhog1* strains. Phosphorylation of BbHCR1 was probed in fungal cells using the 13× Myc-tagged BbHCR1 and Western blotting via Phos-tag SDS-PAGE. BbHCR1 proteins were blotted with anti-myc antibody. Western blot with anti-β-tubulin antibody was used as the internal standard. Proteins were isolated from fungal cells grown under normal (CZB) and CZB containing stressors (75 µM menadione or 1 mM CoCl_2_) at 26°C for 6 h. The relative amounts of the phosphorylated BbHCR1 as compared to their total protein levels for each sample were measured by densitometric analyses of bands using the ImageJ software.

Bioinformatic analysis showed the putative phosphorylation sites of Fus3-MAP kinase (S815) and Hog1-/Fus3-MAP kinase (T865) present in BbHCR1 (Fig. 7A). Yeast two-hybrid tests verified the possible interaction between BbHCR1 with Hog1-MAP kinase (Bbhog1) (33) or Fus3-MAP kinase (Bbmpk1) (34) (Fig. 7B). To reveal the contribution of the predicted phosphorylation sites to BbHCR1 function, serine at position 815 or/and threonine at 865 were mutated to alanine and generated *BbHCR1^S815A^*, *BbHCR1^T865A^*, and *BbHCR1^S815A-T865A^*, which were separately introduced into Δ*BbHCR1* strain. Bioassays via hemocoel injection of conidia revealed that the introduction of *BbHCR1^S815A^* seemed to restore Δ*BbHCR1* to the wild type, while the introduction of *BbHCR1^T865A^* restored Δ*BbHCR1* virulence only by 4.7% (*P* < 0.01). However, the introduction of *BbHCR1^S815A-T865A^* hardly restored the Δ*BbHCR1* virulence (Fig. 7C). These results suggested that BbHCR1 phosphorylation by Fus3-MAP kinase (Bbmpk1) and Hog-MAP kinase (BbHog1) was crucial for its function in fungal virulence, and the contribution of the phosphorylation at site T865 was larger than at site S815.

To verify the effects of Fus3- and Hog1-MAP kinases on the phosphorylation of BbHCR1, PB3::BbHCR1::13myc was separately introduced into wild type, Δ*Bbmpk1* (34) and Δ*BbHog1* (33) strains. Phosphorylation levels of BbHCR1 were examined using Western blots with anti-Myc and horseradish peroxidase (HRP)-conjugated streptavidin following separation of fungal proteins by Phos-tag SDS-PAGE under the normal (1/4 SDY), oxidative (75 µM MND), or low oxygen (1 mM CoCl_2_) conditions. BbHCR1 protein was probed using SDS-PAGE and Western blots. Proteins blotted with anti-β-tubulin antibody were used as the internal standard. The BbHCR1 phosphorylation levels were slightly decreased in the Δ*Bbmpk1* strain under the normal condition (by 18.7%) but observably decreased (by 36.2%–52.6%) under the stress conditions as compared to those in WT (Fig. 7D). Whereas dramatically decreased BbHCR1 phosphorylation levels were detected in Δ*BbHog1* strain as compared to WT either under normal or the stressed conditions (decreased by 50%–72.9%) (Fig. 7E). These results further confirmed the contribution of Fus3- and Hog1-MAP kinases to the phosphorylation of BbHCR1.

## DISCUSSION

Insect pathogenic fungi switch from invasive hyphae to *in vivo* blastospores (hyphal bodies) in the insect hemocoel after penetration of the cuticle, which aids fungal cells to escape the insect immune defense response and colonize insect body cavities. Although some molecules have been characterized in fungal pathogen colonization of insect hemocoel, their regulatory mechanisms are largely unknown (10, 16). Here, we characterized a highly expressed TF (BbHCR1) in *B. bassiana* during the early colonization of insect hemocoel, which regulated the fungal colonization of insect hemocoel. During this process, BbHCR1 targeted CDP activator genes, *brlA* or *abaA,* to control morphological transition from *in vivo* blastospores to hyphae and some virulence-associated genes, and indirectly controlled oosporein and beauverolide biosynthetic gene clusters that are involved in the colonization of insect body cavities (29, 35). Moreover, BbHCR1 was collaboratively regulated by Fus3- and Hog1-MAP kinases via phosphorylation, which was crucial for its regulatory function in insect hemocoel colonization and fungal virulence. These results demonstrated a new regulatory network that controls fungal colonization of insect body cavities.

Significantly decreased/increased virulence was assayed in Δ*BbHCR1*/*BbHCR1*^*OE*^ strains by either topical inoculation (cuticle infection) or injection of conidia into the insect hemocoel (cuticle-bypassing infection) as compared to the wild-type strain; however, no obvious difference was examined in their abilities to penetrate the cuticle using the cicada hind wings to mimic the insect cuticle. These results suggested that the altered virulence of Δ*BbHCR1* and *BbHCR1^OE^* might be due to their different abilities to colonize insect hemocoel. Examination of fungal development revealed that the Δ*BbHCR1* strain generated massive *in vivo* blastospores in insect hemocoel with few hyphae, but *in vivo* blastospores formed from *BbHCR1^OE^*-geminated conidia (germ tubes) rapidly switched to hyphae, suggesting involvement of BbHCR1 in the regulation of morphological transition from blastospores to hyphae *in vivo*. A similar morphological transition was seen in *in vitro* broth. However, reduced/increased conidia production was examined on rich-nutrient 1/4 SDAY in Δ*BbHCR1*/*BbHCR1*^*OE*^ strains, suggesting that BbHCR1 played a distinct role in the regulation of conidiation and blastospore generation. The filamentous fungal conidiation is programmed by a CDP, which comprises the regulators followed by BrlA, AbaA, and WetA that activate target genes to complete conidia formation and maturation (36), all of which are essential for sporulation (conidiation and blastospore generation) and important for hemocoel colonization after the injection of hyphal fragments in *B. bassiana* (35). Our RNA-seq and ChIP-seq data revealed that BbHCR1 acted as a negative regulator of *brlA* and *abaA* during hemocoel colonization since the two genes were significantly upregulated in the Δ*BbHCR1* mutant but downregulated in the overexpression cells. The regulatory role of BbHCR1 in *brlA* and *abaA* expression in insect hemocoel colonization was verified by the disruption of *brlA* or *abaA* in the Δ*BbHCR1* strain and overexpression of them in the *BbHCR1^OE^* strain. Virulence of double mutation (Δ*brlA::*Δ*BbHCR1* or Δ*abaA::*Δ*BbHCR1*) was further decreased as compared to Δ*BbHCR1.* Although the two double overexpression mutants (*brlA^OE^::BbHCR1^OE^* and *brlA^OE^::BbHCR1^OE^*) were more virulent than WT and a difference in virulence was examined in the two strains as compared to the *BbHCR1^OE^* strain, overexpression of *brlA* or *abaA* in the *BbHCR1^OE^* background restored the morphological transition of *BbHCR1^OE^* to the wild type that produced numerous *in vivo* blastospores in insect hemocoel. Our RNA-seq data revealed that several fungal development genes, such as two cell adhesion-associated genes, one conidial cell wall protein gene, and one cell cycle gene displayed opposite expression patterns in insect hemolymph-derived Δ*BbHCR1* and *BbHCR1^OE^* cells. One fungal development and differentiation gene-coding membrane fusion mating protein, FIG1 (32), was identified as a BbHCR1 target, which was significantly downregulated in Δ*BbHCR1* cells. These genes might also contribute to BbHCR1-controlled fungal development in insect hemocoel.

Insect fungal pathogens infect their hosts by direct penetration of the cuticle. After reaching hemocoel, penetrated hyphae differentiate into *in vivo* blastospores that evade the insect immune system and proliferate in the hemocoel (37, 38). However, the Δ*BbHCR1* strain formed numerous blastospores in the insect hemocoel with few hyphae, and unexpectedly, its virulence was significantly decreased. The opposite phenomena were detected in the *BbHCR1^OE^* strain. These results suggested that other virulence- or/and immune-evasion-involved factors might be regulated by BbHCR1 to colonize the insect hemocoel besides control of the morphological transition. Our ChIP-seq and RNA-seq data revealed that four secretory protein genes, encoding a metalloprotease-like protein (BBA_02374) and three hypothetical proteins (BBA_07528 [HP1], BBA_01961 [HP2], and BBA_02460), were identified as BbHCR1 targets, in which BBA_02374 and *HP1* were significantly downregulated in Δ*BbHCR1* cells but upregulated in *BbHCR1^OE^* cells, while other two genes were upregulated in Δ*BbHCR1* cells but downregulated in *BbHCR1^OE^* cells, suggesting these proteins might play opposite roles in fungal colonization of insect hemocoel. Two of the four genes, *HP1* and *HP2*, were selected as representatives and characterized in this study. As expected, disruption of *HP1* significantly decreased *B. bassiana* virulence, while overexpression of *HP2* led to a decrease in fungal virulence. Although the underlying mechanisms were unclear and needed to be revealed in future work, these results suggested that BbHCR1 might activate virulence genes (e.g., *HP1*) but repress virulence repressor-associated genes (e.g., *HP2*) to help colonize insect hemocoel. While the contribution of other BbHCR1 targets (12 of 14) to fungal virulence needs to be determined in the additional work, these results explained the reduced/increased virulence of the Δ*BbHCR1*/*BbHCR1*^*OE*^ strains to some extent. *B. bassiana* produce secondary metabolites, oosporein and beauverolide, during the infection of insects. The former is synthesized after insect body cavity colonization, which contributes to fungal virulence via inhibiting prophenoloxidase activity that facilitates fungal cells to evade the host immune response (29) and to complete the infection cycle via limiting bacterial growth after host death that allows the fungus to maximally use host nutrients (39). The latter also contributes to fungal virulence due to its immunostimulatory and immunosuppressive properties (30). Thus, the two secondary metabolites play important roles in the fungal pathogen colonization of insect body cavities. Our RNA-seq data revealed that biosynthetic gene cluster or genes of oosporein (*Ops1*, *Ops4*, *Ops6,* and *Ops7*) (29) and beauverolide (*besA* and *besB*) (30) were significantly downregulated in Δ*BbHCR1* but upregulated in *BbHCR1^OE^* cells proliferated in the insect blood. These results account for the differences between *BbHCR1^OE^* and Δ*BbHCR1* in the evasion of insect immune responses to some extent (the former but not the latter easily evading insect immune defense responses), affecting their abilities to colonize insect hemocoel.

Our RNA-seq data revealed that some nutrient utilization-involved genes were significantly upregulated in the Δ*BbHCR1* cells but downregulated in the *BbHCR1^OE^* cells, which seemed to be in line with the reduced growth for the *BbHCR1^OE^* strain but more aerial hyphae grown for the Δ*BbHCR1* strain on agar plates. However, rapid growth for *BbHCR1^OE^* cells but delayed growth for Δ*BbHCR1* cells were examined in insect hemocoel, suggesting distinct regulatory roles of BbHCR1 in *in vitro* and *in vivo* growth and development. The contradictory phenomena in *in vitro* and *in vivo* growth of *BbHCR1^OE^* and Δ*BbHCR1* might be caused by their differences in the evasion of the insect immune defense responses examined. Moreover, increased and delayed growth was also examined in *BbHCR1^OE^* and Δ*BbHCR1* strains in broth containing oxidative agent or/and hypoxia stressor, mimicking stress niches in fungal-infected insect hemocoel (27, 28), as compared to WT, respectively, which partially explained their different adaptations to the infected insect hemocoel and their potential role in the control of “oxidative burst” and low oxygen niches during host infection.

The Fus3-MAP kinase-mediated cascades have been shown to control the morphological transition of fungal pathogens, such as the infection structure (appressorium) formation or function, development of infective hyphae, dimorphism, and sporulation, despite the difference in fungal species (10, 40). The pathway also mediates adaptation to oxidative stress derived from insect immune response-generated reactive oxygen species in some fungal species (41). The Hog1-MAP kinase pathway controls osmotic, oxidative, and thermal stress responses, as well as sporulation, viability, and fungal virulence (33), and has been implicated in mediating adaptation to low oxygen niches in fungal-infected insect hemocoel (28). Yeast two-hybrid assays revealed that BbHCR1 interacted with either BbHog1 (Hog1-MAP kinase) (33) or Bbmpk1 (Fus3-MAP kinase) (34), suggesting the involvement of the two kinases in the phosphorylation of BbHCR1. Bioinformatics analysis showed that two predicted phosphorylation sites, S815 and T865, were present in BbHCR1, which were predicted to be special for Fus3-MAP kinase and Fus3-/Hog1-MAP kinase, respectively. The effects of the two kinases on BbHCR1 phosphorylation level were verified using Western blotting assays, in which the phosphorylation levels were significantly decreased in the Δ*Bbmpk1* strain (inactivation of Fus3-MAP kinase) under the low oxygen or oxidative stress conditions as compared to those under normal conditions, suggesting the phosphorylation was at least involved in the adaptation to stress niches in the fungal-infected insect hemocoel. Whereas phosphorylation levels of BbHCR1 were significantly decreased in the Δ*BbHog1* strain (inactivation of Hog1-MAP kinase) under normal, low oxygen, or oxidative stress conditions, suggesting a crucial role of Hog1-MAP kinase in the control of BbHCR1 in fungal development *in vitro* and *in vivo*, as well as adaptation to stress niches in insect hemocoel. Although the introduction of the gene with the mutated phosphorylation site S815 (predicted for Fus3-MAP kinase) seemingly restored the Δ*BbHCR1* virulence, mutation of the site T865 (predicted for either Fus3- or Hog1-MAP kinase) only slightly restored the Δ*BbHCR1* virulence, suggesting that the contribution of phosphorylation at the latter site was greater than at the former site to the BbHCR1 function in fungal colonization of insect hemocoel and virulence. However, mutation of both the sites hardly restored the Δ*BbHCR1* virulence. While it was unclear whether the site S865 was phosphorylated by either Fus3-MAP kinase or Hog1-MAP kinase as a prediction that needs to be determined in the additional work, these results suggested that Fus3-MAP kinase and Hog1-MAP kinase collaboratively regulated BbHCR1 by phosphorylating at the S815 and T865 sites. Moreover, how Fus3-MAP kinase or /and Hog1-MAP kinase affect(s) the function of BbHCR1 via phosphorylation should also be detailed in future work, i.e., controlling the TF internalization into nuclei, transcription activity, as well as binding affinity to different target genes.

Altogether, our results show a TF-mediated network that regulates the fungal colonization of insect body cavities, in which BbHCR1 is collaboratively controlled by Fus3- and Hog1-MAP kinases via phosphorylation, which in turn targets *brlA* and *abaA* and other likely targets for control of morphological transition and virulence factors by tuning the expression of hemocoel colonization-involved immunosuppressive metabolite biosynthetic gene clusters.

## MATERIALS AND METHODS

### Transcription factor co-expression analysis

*B. bassiana* transcription factors were retrieved from Fungal Transcription Factor Database (http://ftfd.snu.ac.kr/download.php) and their gene expression data were extracted from the whole genome expression database that was constructed from 76 deep-sequenced samples covering the growth, development, stress responses, and infection during the life cycle of *B. bassiana* (24). The TF expression data were normalized, and co-expression analysis was performed from the cuticle penetration, hyphal bodies, liquid hyphae, and aerial hyphae database (24). LogCLR and Pearson’s correlation coefficient (PCC) were calculated, and genes with similar expression profiles were clustered. The co-expression network was generated with PCC ≥ 0.6.

### Fungal strains, culture, and gene manipulation

*B. bassiana* wild-type Bb0062 (CGMCC 7.34) and its derived genetically modified strains were cultured as described previously (5). Yeast Y2HGold was used for transcription activation assay and yeast two-hybrid assays. *Escherichia coli* 5α were used for DNA manipulations, and *Agrobacterium tumefaciens* AGL1 were employed for fungal transformation.

The primers for gene manipulations used in this study are given in Table S1. To analyze BbHCR1 gene expression and subcellular localization, *BbHCR1* promoter (2,000 bp) and the full-length gene (5,097 bp, including promoter sequence) were amplified from WT, respectively, and cloned into *Hin*dIII/ *Eco*RV sites of pK2surPB3-GFP (5) to replace the PB3 promoter (*B. bassiana gpd* promoter) (5, 42), generating PBbHCR1::GFP and BbHCR1::GFP constructs. To mark the nucleus, a vector was generated by tagging histone H1 (H1) with a red fluorescent protein. Briefly, *PB3*, *H1,* and *RFP* DNA fragments were separately amplified from plasmids pK2surPB3-GFP, pK2-PB-*H1-eGFP-sur* (43), and p1793 (44), respectively. The three fragments were then fused with overlap PCR and inserted into the *Xba*I/*Hin*dIII sites of pBS-*bar* (45), in which the H1::RFP fusion gene was controlled by the PB3 promoter, forming PB3::H1::RFP. Moreover, BbHCR1 was labeled with 13×Myc and used to detect its phosphorylation using Western blotting and to perform ChIP. Briefly, the PB3 promoter sequence and *BbHCR1* coding region were separately amplified from WT, and 13×Myc and terminator TADH1 sequences were amplified from the plasmid pFA6a-13Myc-KanMX6 (46). The three fragments were fused via overlap PCR, and the resultant product was inserted into *Bam*HI/*Hin*dIII sites of pK2-sur (47), generating PB3::BbHCR1::13myc construct. All those resultant constructs were introduced into WT, BbHCR1::GFP (for PB3::H1::RFP), *BbHCR1* mutant (Δ*BbHCR1*), Δ*Bbhog1,* and Δ*Bbmpk1* strains (for PB3::BbHCR1::13myc) (33, 34) using *A. tumefaciens*-mediated transformation (45).

Target gene (*BbHCR1*, *HP1,* or *HP2*) disruption was performed using *A. tumefaciens*-mediated homologous recombination by replacing the partial gene coding region with the herbicide phosphinothricin resistance *bar* cassette as previously (5). For the disruption of BbHCR1-regulated gene (*abaA* or *brlA*) on *BbHCR1* mutation background, partial coding region of the candidate gene was replaced by the herbicide sulfonylurea resistance gene *sur* cassette via *A. tumefaciens*-mediated homologous recombination (48), generating *ΔabaA::*Δ*BbHCR1* and Δ*brlA::*Δ*BbHCR1*. To reverse complement the mutation strain Δ*BbHCR1*, the wild-type *BbHCR1* (6,711 bp) containing the promoter region were amplified and cloned into *Xba*I/*Hin*dIII sites of pK2-sur (47), which were introduced into Δ*BbHCR1* mutant using *A. tumefaciens*-mediated transformation with the herbicide resistance *sur* gene as the selective marker (5). The correct integration event of the putative gene disruption mutants and reverse complement strains was verified via PCR, while loss or regain of gene transcription was verified by reverse transcription-PCR (RT-PCR).

For overexpression of the genes, coding regions of *BbHCR1*, *HP1*, *HP2, abaA,* and *brlA* were amplified from *B. bassiana*, respectively, and cloned into *Bam*HI/*Eco*RV sites of pK2surPB3-GFP, replacing the GFP sequence and leading to the expression of those target genes controlled by the promoter PB3. The resultant vectors were separately transformed into *B. bassiana* WT (for *BbHCR1*, *HP1,* and *HP2*) and *BbHCR1^OE^* (for *abaA* and *brlA*) strains via *A. tumefaciens*-mediated transformation (45), generating *BbHCR1^OE^*, *HP1^OE^*, *HP2^OE^*, *abaA^OE^::BbHCR1^OE^*, and *brlA^OE^::BbHCR1^OE^* strains.

### Prediction of phosphorylation sites and detection of phosphorylation levels

Phosphorylation sites of BbHCR1 were predicted using Scansite 4.0 (https://scansite4.mit.edu/#scanProtein). The interactions of BbHCR1 with the predicted kinases were verified using yeast two-hybrid tests. The amino acids of phosphorylation sites were mutated to alanine by introducing mutated sites in primers. The *BbHCR1* (total 6,711 bp, containing the promoter region) with introduced mutated sites was amplified and inserted into *Xba*I/*Hin*dIII sites of pK2-sur (47), which were individually introduced into Δ*BbHCR1* using *A. tumefaciens*-mediated transformation with the herbicide resistance *sur* gene as the selective marker (5), generating Δ*BbHCR1::BbHCR1^S815A^,* Δ*BbHCR1::BbHCR1^T865A^*, and Δ*BbHCR1::BbHCR1^S815A-T865A^*. The contribution of phosphorylation sites to the BbHCR1 function was investigated by comparison of virulence between the reverse complement strains with the WT gene and the mutated site(s)-containing gene(s) and WT. All the primers are shown in Table S1.

For the detection of BbHCR1 and its phosphorylated protein, Western blotting was performed. Briefly, fungal cells harboring the BbHCR1::13 myc fusion gene (in WT, Δ*Bbhog1*, and Δ*Bbmpk1*) from 1/4 SDY were cultured at 26°C for 60 h (normal condition) or in the Czapek-Dox Broth (BD Difco) containing stressors (75 µM menadione or 1 mM CoCl_2_) for 6 h at 26°C. Fungal cells were collected and immediately homogenized in liquid nitrogen for protein extraction. Proteins were extracted from ca*.* 0.1 g samples using 600 µL of cell lysis buffer [0.4 M NH_4_(SO_4_)_2_, 10 mM MgCl_2_, 10% glycerol, and 2 mM β-mercaptoethanol in 0.2 M Tris-HCl (pH 8.0)] by adding protease inhibitors phenylmethanesulfonyl fluoride (0.2 mg/mL), cOmplete Tablets EDTA-free (Roche) (0.98 mg/mL), and protein phosphatase inhibitor, PhosSTOP (Roche) (4.7 mg/mL). Protein was quantified using the BCA Assay Kit (GENEray), and 60 µg proteins was separated by 12.5% SDS-PAGE and Phos-tag SDS-PAGE (including 50 µM Phos-tag [Wako Pure Chemical Industries] and 100 µM MnCl_2_). The BbHCR1 and its phosphorylated protein were blotted with anti-myc tag mouse polyclonal antibody (Thermo Fisher Scientific Inc.) and HRP-conjugated rabbit anti-mouse secondary antibody (Thermo Fisher Scientific Inc.). Anti-β-tubulin antibody (Sigma) was used as the internal standard. The gray values of the protein bands were estimated in Western blots using ImageJ program (49), and the relative phosphorylation levels of BbHCR1 were estimated by comparison of phosphorylated protein bands (from Phos-tag SDS-PAGE) to total BbHCR1 protein bands (from SDS-PAGE).

### Gene expression analysis, transcription activation, and yeast two-hybrid assays

Gene expression analysis was performed using RT-PCR, RT-qPCR, and/ or by the detection of GFP fluorescence signals in fungal cells of the PBHCR1::GFP strain. For RT-PCR and RT-qPCR, 300 ng of total RNA was reverse-transcribed using an oligo(dT)-primed cDNA synthesis kit with gDNA Eraser (Aidlab, Beijing). The first-strand cDNA was used as the template for PCR. RT-qPCR analysis was conducted using ChamQ Universal SYBR qPCR Master Mix (Vazyme, Nanjing). RT-PCR and RT-qPCR were performed by using 18S rRNA (BBA_07911) as a reference gene as described previously (5). Loss or regain of target gene transcription in the gene disruption or complement strains was analyzed using RT-PCR from 1/4 SDY cultures for 60 h. The transcription level of *BbHCR1* in the gene overexpression strains was evaluated using RT-qPCR from 1/4 SDY cultures for 60 h. Transcription levels of *BbHCR1* in WT at different infection stages (*in vivo*), saprophytic hyphae (*in vitro*), and stressed by low oxygen or oxidative agents were evaluated using RT-qPCR and by the detection of GFP fluorescence signals in the PBHCR1::GFP cells. To prepare fungal cells during insect infection, 2 µL conidial suspension (10^7^ conidia/mL) was inoculated in the last-instar *Galleria mellonella* larvae by injection at the second proleg. Fungal cells during insect infection were separately collected by blooding at 48 and 72 h post-injection and dissecting the tissues at 84 and 96 h after inoculation. The saprophytic hyphae (*in vitro*), including aerial and submerged hyphae, were prepared from 3-day 1/4 SDAY (agar) or 1/4 SDY (broth) with aeration at 26°C as described previously (5). The fungal cells stressed by LO or oxidative stresses were prepared by inoculation of 1/4 SDY cultures (for 60 h) in the Czapek-Dox Broth containing 1 mM CoCl_2_ (a hypoxia-mimicking agent) (50, 51) or oxidative stressors (75 µM MND) for 6 h. All primers for gene expression analysis are shown in Table S1.

For the transcriptional activation test, full-length *BbHCR1* cDNA was amplified from WT and cloned into the *Eco*RI/*Bam*HI sites of the yeast vector pGBKT7 (Clontech) under the *GAL4* promoter to generate plasmid pGBKT7-BbHCR1, which was transformed into the yeast Y2HGold strain. The putative transformants were grown on the medium SD/-Trp and SD/-Trp with 0.35 µg/mL AbA (Aureobasidin A) and X-α-Gal. The positive control yeast was made by transforming with the pGBKT7 vector containing the GAL4 activation domain and the GAL4-binding domain, while the negative control yeast was transformed with the blank vector harboring the GAL4-binding domain.

Yeast two-hybrid assays were used to verify the interaction between BbHCR1 and the predicted kinase(s) that might phosphorylate BbHCR1. Briefly, cDNAs of the predicted kinases, Fus3-MAP kinase (Bbmpk1) (34) and Hog1-MAP kinase (Bbhog1) (33) genes, were separately amplified from *B. bassiana* and individually inserted into *Eco*RI/*Bam*HI sites of the plasmid pGBKT7 as the prey carriers. The *BbHCR1* cDNA was amplified and cloned into *Eco*RI/*Bam*HI sites of the plasmid pGADT7 as the bait carrier. The resultant plasmids were co-transformed into Y2HGold cells. The resultant strains were verified by PCR and cultured on SD/-Leu/-Trp and SD/-Leu/-Trp/-His/-Ade auxotroph medium containing X-α-Gal and AbA (0.35 µg/mL), respectively. Y2HGold cells harboring pGADT7-T and pGBKT7-53 or pGADT7-T and pGBKT7-Lam were used as positive or negative control, respectively. All the primer pairs are shown in Table S1.

### Insect bioassays and detection of fungal development in the insect hemocoel

Insect bioassays were performed using last-instar *G. mellonella* larvae either by topical application of conidia on the insect cuticle or direct injection of conidia into the insect hemocoel as described previously (5). For the topical application, 1 mL of conidial suspension (10^7^ conidia/mL) was inoculated by spray on the larvae surface. For injection assays, 2 µL of conidial suspension (10^5^ conidia/mL) was microinjected into the larvae via the second proleg. Since the capacities of Δ*brlA::*Δ*BbHCR1* and Δ*abaA::*Δ*BbHCR1* strains to sporulate were completely lost, the mycelial fragments were prepared from their 1/4 SDY cultures for 3 days with ultrasonication (AMPL 25%, 6 s on and 6 s off for 10 min). The fragments were suspended in 0.05% Tween-80 and adjusted to a concentration of 20 mg fresh hyphae/mL, which were used for insect bioassay via injection (3 µL per larvae). Controls were treated with 0.05% Tween-80. All experiments were repeated three times with 90 larvae per replicate. Mortality was recorded every 12 h.

A subset of larvae in microinjection bioassays were bled at 24, 36, 48, 60, and 72 h post-treatment to microscopically monitor the fungal development. Fungal cells were quantified using qPCR analysis by amplification of the 18S rRNA sequence as previously described (5). For the tissue frozen section, the infected larvae were quickly frozen with liquid nitrogen at 60 h post-injection and sectioned with Microtome Cryostat (Thermo), in which the fungal cells in tissues were stained with lactic acid phenol cotton blue solution for microscopic observation.

### RNA sequencing and ChIP sequencing

RNAs were isolated from WT, Δ*BbHCR1,* and *BbHCR1^OE^* cells that were proliferated in the *G. mellonella* larvae hemocoel for 48 h after microinjection of 2 µL of conidial suspension (5 × 10^7^ conidia /mL) or cultured in 1/4 SDY for 3 days and sequenced using the Illumina HiSeq 2000 platform (Novogene, Beijing, China). Briefly, the inoculated larvae were bled and mixed with the anticoagulant solution as described previously (38). The mixtures were resuspended in sterile water for breaking the hemocytes (due to low osmotic pressure) and centrifuged to remove hemocyte and hemolymph debris, which were repeated three times. After examination of fungal cells under a microscope, the pellets were used for RNA isolation. Reads were mapped to the *B. bassiana* 2860 genome (52) after the removal of adaptor tags, low-quality tags, and tags with only a single copy. Differentially expressed genes were identified between Δ*BbHCR1* or *BbHCR1^OE^* and WT RNA-seq libraries using the number of fragments per kilobase of exon region per million mappable reads (FPKM). A minimum of twofold expressional difference (i.e., log_2_ FoldChange < −1.0 or > 1.0) in the paired libraries was used as a standard to judge each DEG at the false discovery rate of 0.05 or less (53). Sequencing was performed two times with different batches of fungal cells, and the reliability was assessed by Pearson’s correlation coefficients (*r*) based on FPKM values. DEGs were classified and annotated using KEGG pathway enrichment analysis.

ChIP was performed using the *B. bassiana* PB3::BbHCR1::13myc strain, in which 13 Myc-tagged *BbHCR1* controlled by the constitutive promoter PB3 was expressed in the Δ*BbHCR1* strain. The resultant strain restored phenotypes of Δ*BbHCR1* to wild type, including growth and virulence. ChIP was performed as described previously (54) using Pierce anti-c-myc magnetic beads (ThermoFisher) with fungal cells from 1/4 SDY cultures at 26°C for 60 h with aeration (200 rpm). The purified DNA from the immunocomplexes was used for ChIP sequencing with the Illumina HiSeq 2000 platform (Novogene, Beijing, China) for 76 bp paired-end sequencing. After cleaning contaminating adaptors using trimmomatic software (55), reads were aligned to the *B. bassiana* 2860 genome (52). Peaks with a *P* value of <0.005 were chosen as candidate binding sites and targeted genes were identified if peaks were located within their promoter regions (about 2.0 kb). The motifs within ChIP-seq peaks were analyzed using an online motif predictor, the Multiple Em for Motif Elicitation (https://meme-suite.org/meme/) tool (56). The identified target genes of BbHCR1 were verified using electrophoretic mobility shift assay. Briefly, the predicted DNA-binding domain of BbHCR1 (411 bp) was amplified and cloned into pET28a, which was introduced into *E. coli* BL21 to express His-tagged fusion protein following standard protocol. Proteins were purified using a Mag-Beads His-Tag Protein Purification Kit (Sangon, China). The promoter fragments containing predicted BbHCR1-binding motifs of the target genes (472, 322, 328, and 369 bp for *brlA*, *abaA*, *HP1,* and *HP2*, respectively) were amplified (primers are shown in Table S1) and used for EMSA assays with His-tagged DNA-binding domain of BbHCR1. The ability of BbHCR1 to bind promoter regions of the target genes was detected in gels using the LightShift Chemiluminescent EMSA Kit (Thermo Fisher, USA) following the manufacturer’s instructions.

### Data analysis

The survival data in insect bioassays were plotted as Kaplan-Meyer curves, and a log-rank test was used to analyze the difference between groups with the GraphPad Prism 8.0 program. The mean lethal time to kill 50% of insects (LT_50_) was calculated using the SPSS 17.0 program. The differences between different groups were tested using a one-way analysis of variance and LSD test.

## Data Availability

Sequencing data have been deposited in the SRA under the accession codes PRJNA1029189, PRJNA973851 (RNA sequences from normal cultures and insect hemolymph-derived hyphal bodies, respectively), and PRJNA1050806 (ChIP sequences). Other relevant data supporting the findings of this study are available in this article and its associated supplemental material.

## AFTER EPUB

*[This article was published on 15 May 2024 with an error in the Importance. The text was corrected in the current version, posted on 3 June 2024.]*

## SUPPLEMENTAL MATERIAL

Supplemental material mbio.03504-23-s0001.docx Table S1 caption and Figures S1 to S9.

Table S1 mbio.03504-23-s0002.docx Primers used in this study.

ASM does not own the copyrights to Supplemental Material that may be linked to, or accessed through, an article. The authors have granted ASM a non-exclusive, world-wide license to publish the Supplemental Material files. Please contact the corresponding author directly for reuse.

## References

[1] Wang B, Kang Q, Lu Y, Bai L, Wang C. 2012. Unveiling the biosynthetic puzzle of destruxins in Metarhizium species. Proc Natl Acad Sci U S A 109:1287–1292. doi:10.1073/pnas.1115983109 22232661 PMC3268274

[2] Doehlemann G, Ökmen B, Zhu W, Sharon A. 2017. Plant pathogenic fungi. Microbiol Spectr 5. doi:10.1128/microbiolspec.FUNK-0023-2016 PMC11687436 28155813

[3] Köhler JR, Hube B, Puccia R, Casadevall A, Perfect JR. 2017. Fungi that infect humans. Microbiol Spectr 5. doi:10.1128/microbiolspec.FUNK-0014-2016 PMC11687496 28597822

[4] Childers DS, Avelar GM, Bain JM, Pradhan A, Larcombe DE, Netea MG, Erwig LP, Gow NAR, Brown AJP. 2020. Epitope shaving promotes fungal immune evasion. mBio 11:e00984-20. doi:10.1128/mBio.00984-20 32636248 PMC7343991

[5] Lu Z, Deng J, Wang H, Zhao X, Luo Z, Yu C, Zhang Y. 2021. Multifunctional role of a fungal pathogen-secreted laccase 2 in evasion of insect immune defense. Environ Microbiol 23:1256–1274. doi:10.1111/1462-2920.15378 33393158

[6] Yuan Y, Huang W, Chen K, Ling E. 2020. Beauveria bassiana ribotoxin inhibits insect immunity responses to facilitate infection via host translational blockage. Dev Comp Immunol 106:103605. doi:10.1016/j.dci.2019.103605 31904434

[7] Yang M, Solis NV, Marshall M, Garleb R, Zhou T, Wang D, Swidergall M, Pearlman E, Filler SG, Liu H. 2022. Control of β-glucan exposure by the endo-1,3-glucanase Eng1 in Candida albicans modulates virulence. PLoS Pathog 18:e1010192. doi:10.1371/journal.ppat.1010192 34995333 PMC8775328

[8] Wang H, Lu Z, Keyhani NO, Deng J, Zhao X, Huang S, Luo Z, Jin K, Zhang Y. 2023. Insect fungal pathogens secrete a cell wall-associated glucanase that acts to help avoid recognition by the host immune system. PLoS Pathog 19:e1011578. doi:10.1371/journal.ppat.1011578 37556475 PMC10441804

[9] Karkowska-Kuleta J, Rapala-Kozik M, Kozik A. 2009. Fungi pathogenic to humans: molecular bases of virulence of Candida albicans, Cryptococcus neoformans and Aspergillus fumigatus. Acta Biochim Pol 56:211–224. 19543556

[10] Hong S, Shang J, Sun Y, Tang G, Wang C. 2024. Fungal infection of insects: molecular insights and prospects. Trends Microbiol 32:302–316. doi:10.1016/j.tim.2023.09.005 37778923

[11] Nobile CJ, Solis N, Myers CL, Fay AJ, Deneault JS, Nantel A, Mitchell AP, Filler SG. 2008. Candida albicans transcription factor Rim101 mediates pathogenic interactions through cell wall functions. Cell Microbiol 10:2180–2196. doi:10.1111/j.1462-5822.2008.01198.x 18627379 PMC2701370

[12] Beyhan S, Gutierrez M, Voorhies M, Sil A. 2013. A temperature-responsive network links cell shape and virulence traits in a primary fungal pathogen. PLoS Biol 11:e1001614. doi:10.1371/journal.pbio.1001614 23935449 PMC3720256

[13] Mandel MA, Beyhan S, Voorhies M, Shubitz LF, Galgiani JN, Orbach MJ, Sil A. 2022. The WOPR family protein Ryp1 is a key regulator of gene expression, development, and virulence in the thermally dimorphic fungal pathogen Coccidioides posadasii. PLoS Pathog 18:e1009832. doi:10.1371/journal.ppat.1009832 35385558 PMC9015156

[14] Pongpom M, Liu H, Xu W, Snarr BD, Sheppard DC, Mitchell AP, Filler SG. 2015. Divergent targets of Aspergillus fumigatus AcuK and AcuM transcription factors during growth in vitro versus invasive disease. Infect Immun 83:923–933. doi:10.1128/IAI.02685-14 25534941 PMC4333448

[15] Liu H, Xu W, Bruno VM, Phan QT, Solis NV, Woolford CA, Ehrlich RL, Shetty AC, McCraken C, Lin J, Bromley MJ, Mitchell AP, Filler SG. 2021. Determining Aspergillus fumigatus transcription factor expression and function during invasion of the mammalian lung. PLoS Pathog 17:e1009235. doi:10.1371/journal.ppat.1009235 33780518 PMC8031882

[16] Zhang X, Meng Y, Huang Y, Zhang D, Fang W. 2021. A novel cascade allows Metarhizium robertsii to distinguish cuticle and hemocoel microenvironments during infection of insects. PLoS Biol 19:e3001360. doi:10.1371/journal.pbio.3001360 34347783 PMC8366996

[17] Todd RB, Zhou M, Ohm RA, Leeggangers HACF, Visser L, de Vries RP. 2014. Prevalence of transcription factors in ascomycete and basidiomycete fungi. BMC Genomics 15:214. doi:10.1186/1471-2164-15-214 24650355 PMC3998117

[18] Wang C, Feng MG. 2014. Advances in fundamental and applied studies in China of fungal biocontrol agents for use against arthropod pests. Biol Control 68:129–135. doi:10.1016/j.biocontrol.2013.06.017

[19] Ortiz-Urquiza A, Keyhani NO. 2015. Stress response signaling and virulence: insights from entomopathogenic fungi. Curr Genet 61:239–249. doi:10.1007/s00294-014-0439-9 25113413

[20] Ortiz-Urquiza A, Keyhani NO. 2013. Action on the surface: entomopathogenic fungi versus the insect cuticle. Insects 4:357–374. doi:10.3390/insects4030357 26462424 PMC4553469

[21] Pedrini N, Ortiz-Urquiza A, Huarte-Bonnet C, Fan Y, Juárez MP, Keyhani NO. 2015. Tenebrionid secretions and a fungal benzoquinone oxidoreductase form competing components of an arms race between a host and pathogen. Proc Natl Acad Sci U S A 112:E3651–E3660. doi:10.1073/pnas.1504552112 26056261 PMC4507192

[22] Lu H-L, St Leger RJ. 2016. Insect immunity to entomopathogenic fungi. Adv Genet 94:251–285. doi:10.1016/bs.adgen.2015.11.002 27131327

[23] Ortiz-Urquiza A, Keyhani NO. 2016. Molecular genetics of Beauveria Bassiana infection of insects. Adv Genet 94:165–249. doi:10.1016/bs.adgen.2015.11.003 27131326

[24] Huang S, Zhao X, Luo Z, Tang X, Zhou Y, Keyhani N, Zhang Y. 2023. Fungal co-expression network analyses identify pathogen gene modules associated with host insect invasion. Microbiol Spectr 11:e0180923. doi:10.1128/spectrum.01809-23 37656157 PMC10581046

[25] Horton P, Park KJ, Obayashi T, Fujita N, Harada H, Adams-Collier CJ, Nakai K. 2007. WoLF PSORT: protein localization predictor. Nucleic Acids Res 35:W585–W587. doi:10.1093/nar/gkm259 17517783 PMC1933216

[26] Deng J, Lu Z, Wang H, Li N, Song G, Zhu Q, Sun J, Zhang Y. 2022. A secretory phospholipase A2 of a fungal pathogen contributes to lipid droplet homeostasis, assimilation of insect-derived lipids, and repression of host immune responses. Insect Sci 29:1685–1702. doi:10.1111/1744-7917.13029 35276754

[27] He Z, Zhang S, Keyhani NO, Song Y, Huang S, Pei Y, Zhang Y. 2015. A novel mitochondrial membrane protein, Ohmm, limits fungal oxidative stress resistance and virulence in the insect fungal pathogen Beauveria bassiana. Environ Microbiol 17:4213–4238. doi:10.1111/1462-2920.12713 25403093

[28] He Z, Zhao X, Gao Y, Keyhani NO, Wang H, Deng J, Lu Z, Kan Y, Luo Z, Zhang Y. 2020. The fungal mitochondrial membrane protein, BbOhmm, antagonistically controls hypoxia tolerance. Environ Microbiol 22:2514–2535. doi:10.1111/1462-2920.14910 31894607

[29] Feng P, Shang Y, Cen K, Wang C. 2015. Fungal biosynthesis of the bibenzoquinone oosporein to evade insect immunity. Proc Natl Acad Sci U S A 112:11365–11370. doi:10.1073/pnas.1503200112 26305932 PMC4568701

[30] Yin Y, Chen B, Song S, Li B, Yang X, Wang C. 2020. Production of diverse beauveriolide analogs in closely related fungi: a rare case of fungal chemodiversity. mSphere 5:e00667-20. doi:10.1128/mSphere.00667-20 32878933 PMC7471007

[31] Ren Y, Zhang C, Chen Z, Lu L. 2021. The heterotrimeric transcription factor CCAAT-binding complex and Ca^2+^-CrzA signaling reversely regulate the transition between fungal hyphal growth and asexual reproduction. mBio 12:e0300721. doi:10.1128/mBio.03007-21 34781745 PMC8593669

[32] Cavinder B, Trail F. 2012. Role of Fig1, a component of the low-affinity calcium uptake system, in growth and sexual development of filamentous fungi. Eukaryot Cell 11:978–988. doi:10.1128/EC.00007-12 22635922 PMC3416067

[33] Zhang Y, Zhao J, Fang W, Zhang J, Luo Z, Zhang M, Fan Y, Pei Y. 2009. Mitogen-activated protein kinase hog1 in the entomopathogenic fungus Beauveria bassiana regulates environmental stress responses and virulence to insects. Appl Environ Microbiol 75:3787–3795. doi:10.1128/AEM.01913-08 19363067 PMC2687298

[34] Zhang Y, Zhang J, Jiang X, Wang G, Luo Z, Fan Y, Wu Z, Pei Y. 2010. Requirement of a mitogen-activated protein kinase for appressorium formation and penetration of insect cuticle by the entomopathogenic fungus Beauveria bassiana. Appl Environ Microbiol 76:2262–2270. doi:10.1128/AEM.02246-09 20139313 PMC2849248

[35] Zhang AX, Mouhoumed AZ, Tong SM, Ying SH, Feng MG. 2019. BrlA and AbaA govern virulence-required dimorphic switch, conidiation, and pathogenicity in a fungal insect pathogen. mSystems 4:e00140-19. doi:10.1128/mSystems.00140-19 31289140 PMC6616149

[36] Li F, Shi HQ, Ying SH, Feng MG. 2015. WetA and VosA are distinct regulators of conidiation capacity, conidial quality, and biological control potential of a fungal insect pathogen. Appl Microbiol Biotechnol 99:10069–10081. doi:10.1007/s00253-015-6823-7 26243054

[37] Lewis MW, Robalino IV, Keyhani NO. 2009. Uptake of the fluorescent probe FM4-64 by hyphae and haemolymph-derived in vivo hyphal bodies of the entomopathogenic fungus Beauveria bassiana. Microbiology (Reading) 155:3110–3120. doi:10.1099/mic.0.029165-0 19542008

[38] Wanchoo A, Lewis MW, Keyhani NO. 2009. Lectin mapping reveals stage-specific display of surface carbohydrates in in vitro and haemolymph-derived cells of the entomopathogenic fungus Beauveria bassiana. Microbiology (Reading) 155:3121–3133. doi:10.1099/mic.0.029157-0 19608611

[39] Fan Y, Liu X, Keyhani NO, Tang G, Pei Y, Zhang W, Tong S. 2017. Regulatory cascade and biological activity of Beauveria bassiana oosporein that limits bacterial growth after host death. Proc Natl Acad Sci U S A 114:E1578–E1586. doi:10.1073/pnas.1616543114 28193896 PMC5338512

[40] Xu JR. 2000. Map kinases in fungal pathogens. Fungal Genet Biol 31:137–152. doi:10.1006/fgbi.2000.1237 11273677

[41] Zhao X, Jiang Y, Wang H, Lu Z, Huang S, Luo Z, Zhang L, Lv T, Tang X, Zhang Y. 2023. Fus3/Kss1-MAP kinase and Ste12-like control distinct biocontrol-traits besides regulation of insect cuticle penetration via phosphorylation cascade in a filamentous fungal pathogen. Pest Manag Sci 79:2611–2624. doi:10.1002/ps.7446 36890107

[42] Liao X, Fang W, Zhang Y, Fan Y, Wu X, Zhou Q, Pei Y. 2008. Characterization of a highly active promoter, PBbgpd, in Beauveria bassiana. Curr Microbiol 57:121–126. doi:10.1007/s00284-008-9163-3 18443858

[43] Zhang J, Jin K, Xia Y. 2017. Contributions of β-tubulin to cellular morphology, sporulation and virulence in the insect-fungal pathogen, Metarhizium acridum*.* Fungal Genet Biol 103:16–24. doi:10.1016/j.fgb.2017.03.005 28336393

[44] Ren H, Li X, Li Y, Li M, Sun J, Wang F, Zeng J, Chen Y, Wang L, Yan X, Fan Y, Jin D, Pei Y. 2022. Loss of function of VdDrs2, a P4-ATPase, impairs the toxin secretion and microsclerotia formation, and decreases the pathogenicity of Verticillium dahliae. Front Plant Sci 13:944364. doi:10.3389/fpls.2022.944364 36072318 PMC9443849

[45] Ma JC, Zhou Q, Zhou YH, Liao XG, Zhang YJ, Jin D, Pei Y. 2009. The size and ratio of homologous sequence to non-homologous sequence in gene disruption cassette influences the gene targeting efficiency in Beauveria bassiana. Appl Microbiol Biotechnol 82:891–898. doi:10.1007/s00253-008-1844-0 19148636

[46] Longtine MS, Mckenzie III A, Demarini DJ, Shah NG, Wach A, Brachat A, Philippsen P, Pringle JR. 1998. Additional modules for versatile and economical PCR-based gene deletion and modification in Saccharomyces cerevisiae. Yeast 14:953–961. doi:10.1002/(SICI)1097-0061(199807)14:10<953::AID-YEA293>3.0.CO;2-U 9717241

[47] Wang H, He Z, Luo L, Zhao X, Lu Z, Luo T, Li M, Zhang Y. 2018. An aldo-keto reductase, Bbakr1, is involved in stress response and detoxification of heavy metal chromium but not required for virulence in the insect fungal pathogen, Beauveria bassiana. Fungal Genet Biol 111:7–15. doi:10.1016/j.fgb.2018.01.001 29305969

[48] Yue Y, Deng J, Wang H, Lv T, Dou W, Jiao Y, Peng X, Zhang Y. 2023. Two secretory T2 RNases act as cytotoxic factors contributing to the virulence of an insect fungal pathogen. J Agric Food Chem 71:7069–7081. doi:10.1021/acs.jafc.3c01617 37122240

[49] Jensen EC. 2013. Quantitative analysis of histological staining and fluorescence using ImageJ. Anat Rec 296:378–381. doi:10.1002/ar.22641 23382140

[50] Lee H, Bien CM, Hughes AL, Espenshade PJ, Kwon-Chung KJ, Chang YC. 2007. Cobalt chloride, a hypoxia-mimicking agent, targets sterol synthesis in the pathogenic fungus Cryptococcus neoformans. Mol Microbiol 65:1018–1033. doi:10.1111/j.1365-2958.2007.05844.x 17645443

[51] Ingavale SS, Chang YC, Lee H, McClelland CM, Leong ML, Kwon-Chung KJ. 2008. Importance of mitochondria in survival of Cryptococcus neoformans under low oxygen conditions and tolerance to cobalt chloride. PLoS Pathog 4:e1000155. doi:10.1371/journal.ppat.1000155 18802457 PMC2528940

[52] Xiao G, Ying S-H, Zheng P, Wang Z-L, Zhang S, Xie X-Q, Shang Y, St Leger RJ, Zhao G-P, Wang C, Feng M-G. 2012. Genomic perspectives on the evolution of fungal entomopathogenicity in Beauveria bassiana. Sci Rep 2:483. doi:10.1038/srep00483 22761991 PMC3387728

[53] Audic S, Claverie JM. 1997. The significance of digital gene expression profiles. Genome Res 7:986–995. doi:10.1101/gr.7.10.986 9331369

[54] Wang Y, An C, Zhang X, Yao J, Zhang Y, Sun Y, Yu F, Amador DM, Mou Z. 2013. The Arabidopsis elongator complex subunit2 epigenetically regulates plant immune responses. Plant Cell 25:762–776. doi:10.1105/tpc.113.109116 23435660 PMC3608791

[55] Lohse M, Bolger AM, Nagel A, Fernie AR, Lunn JE, Stitt M, Usadel B. 2012. RobiNA: a user-friendly, integrated software solution for RNA-seq-based transcriptomics. Nucleic Acids Res 40:W622–W627. doi:10.1093/nar/gks540 22684630 PMC3394330

[56] Bailey TL, Boden M, Buske FA, Frith M, Grant CE, Clementi L, Ren J, Li WW, Noble WS. 2009. MEME SUITE: tools for motif discovery and searching. Nucleic Acids Res 37:W202–W208. doi:10.1093/nar/gkp335 19458158 PMC2703892

