# Supplementary material for: A transcription factor-mediated regulatory network controls fungal pathogen colonization of insect body cavities
Source: mBio. 2024 May 15;15(6):e03504-23. doi: 10.1128/mbio.03504-23 (PMC11237471; doi:10.1128/mbio.03504-23)
Supplement: Supplemental material — Table S1 caption and Figures S1 to S9. [file mbio.03504-23-s0001.docx]

**Supplementary Material**

**Deng J, Huang S, Kan Y, Song Y, Zhao X, Li N, Yao X, Luo Z., Zhang Y. (2024) A transcription factor-mediated regulatory network controls a fungal pathogen colonization of insect body cavities**

# Supplementary Tables

**Table S1 Primers used in this study**

# Supplementary Figures

**
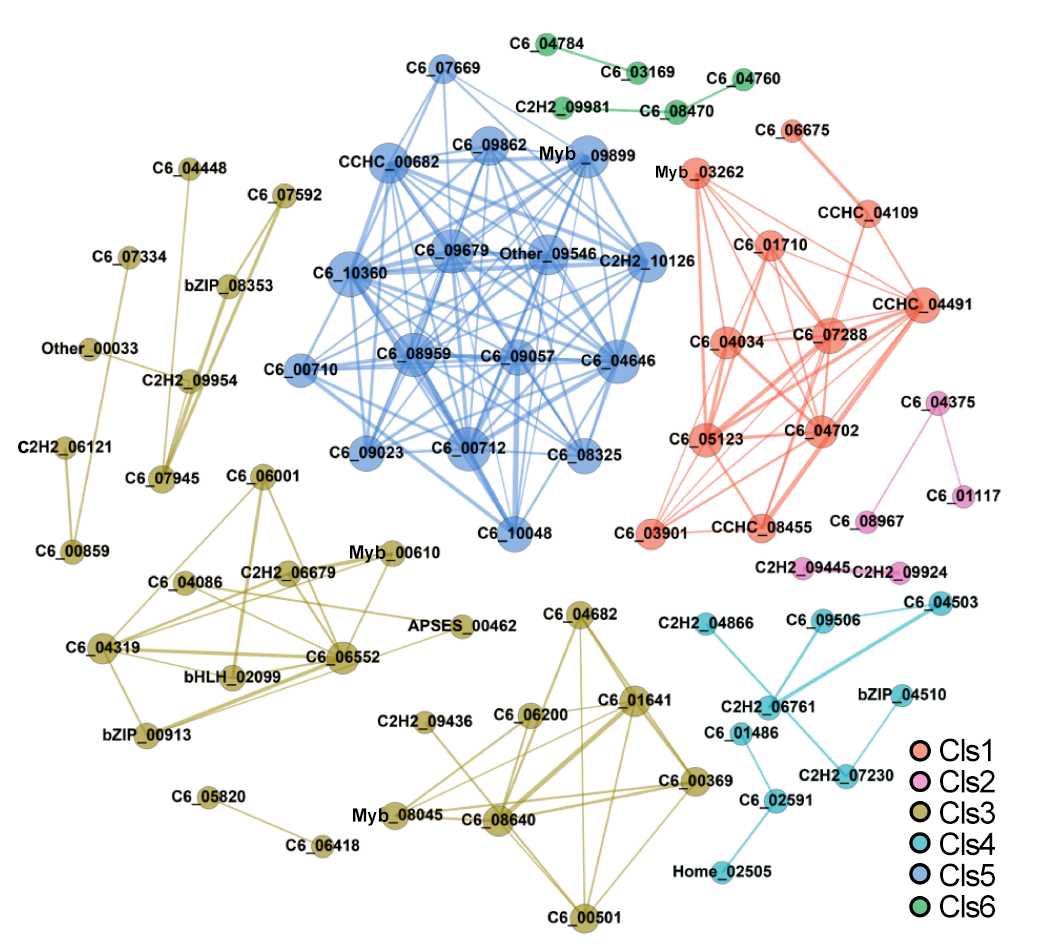
**

**Fig S1. Co-expression network of *B. bassiana* TF genes.** Positive gene-gene PCC (Pearson’s correlation coefficient) correlations of over 0.6 were used for visualization. Size of node represented connection counts, and connections between genes indicated a co-expression relationship.


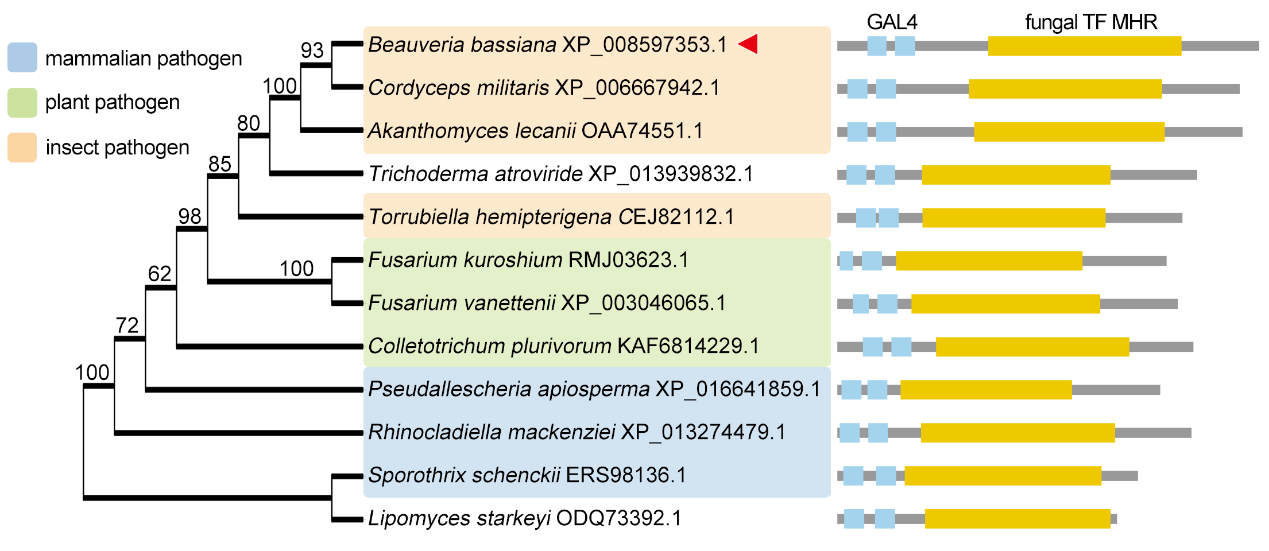


**Fig S2. Phylogenetic analysis of BbHCR1 homologues**. Homologues of BbHCR1 (XP_008597353.1) were searched through the UniProt database (https://www.uniprot.org/). Domains were predicted from each orthologue sequence at https://www.ebi.ac.uk/interpro/. Phylogenetic analysis was performed with a Neighbor-joining method in MEGA6. The red triangle indicated BbHCR1. Bootstrap values of 500 replications were shown at nodes. The structural diagram of BbHCR1 and homologues were indicated, in which the blue and yellow rectangles indicated the GAL4 and fungal TF MHR domains, respectively.

**
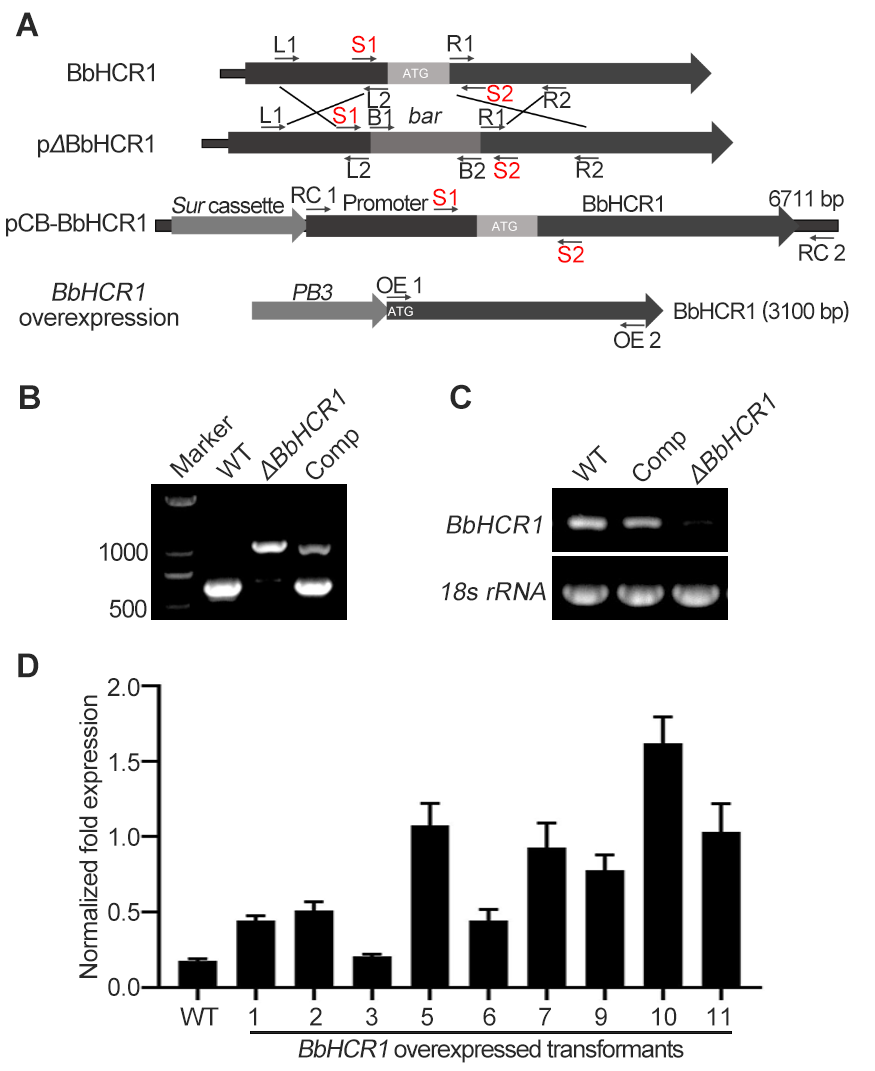
**

**Fig S3. Targeted gene disruption, complementation and overexpression.** **(A)** *BbHCR1* locus and gene replacement vector pΔBbHCR1, overexpression vector and complementation vector pCB-BbHCR1. Homologous recombination (cross over event marked by ‘X’) resulting in a region of *BbHCR1* was replaced by the *bar* cassette. Primers used for generation of the vectors and for verification of transformants with PCR were indicated in the constructures. **(B-C)** PCR (with primers pair S1/S2) and RT-PCR analysis of WT, *ΔBbHCR1*, and *BbHCR1* complementation (Comp) strains. **(D)** RT-qPCR determination of *BbHCR1* transcript levels in WT and *BbHCR1* overexpression (*BbHCR1^OE^*) strains with *18S rRNA* as a reference gene.

**
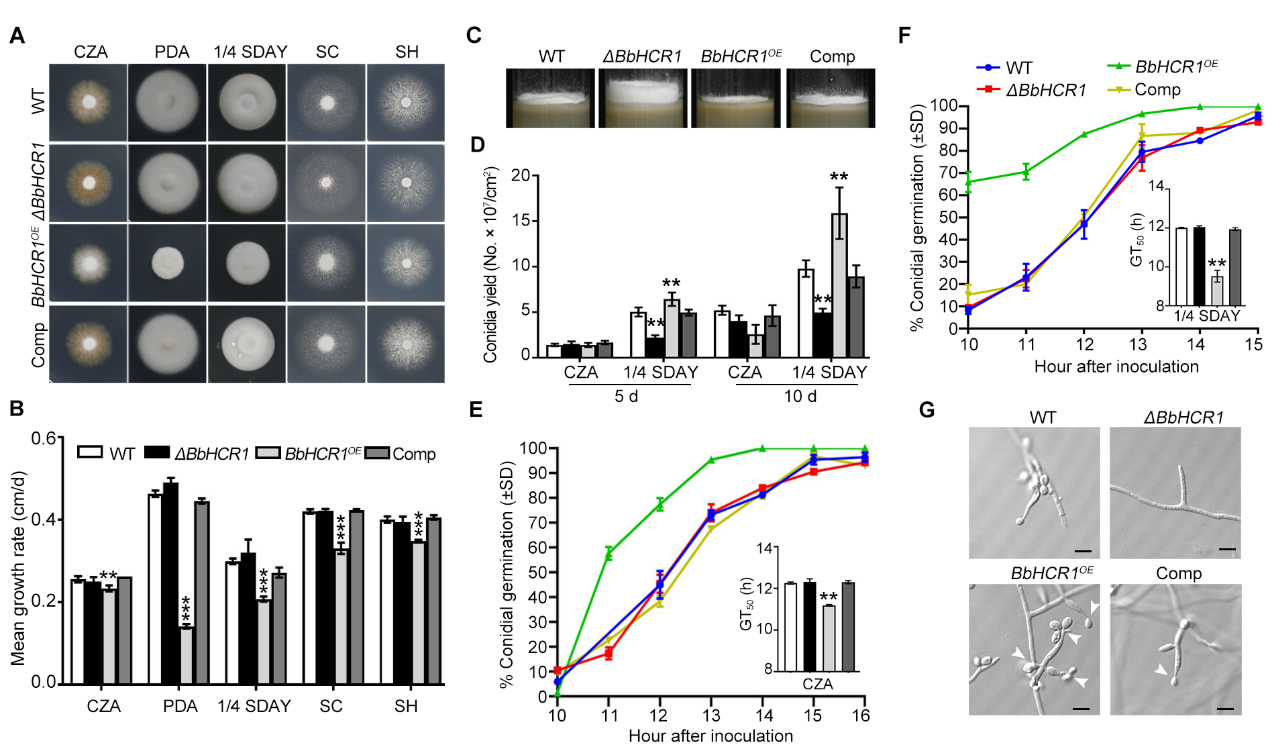
**

**Fig S4. Fungal growth, conidiation and conidial germination on agar plates.** **(A)** Colony grown for 7 days at 26^o^C at indicated agar plates. Fungal strains were inoculated by dropping 2 μl of conidial suspension (10^7^ conidia/ml) on the center of 1/4 SDAY (1:4-diluted Sabouraud dextrose agar supplemented with 1% (w/v) yeast extract), PDA, CZA and basic salt broth supplemented with 5 ml/l silkworm hemolymph (SH) and 1.67 g/l cuticle (SC). **(B)** Colony growth rates calculated based on colony growth in dimeters using linear regression method. **(C)** Aerial hypha development in 1/4 SDAY in test tubes for 7 days at 26^o^C. **(D)** Conidial yield per cm^2^ on CZA and 1/4 SDAY at indicated time. **(E-F)** Conidial germination and calculated the mean germination time (GT_50_) on CZA and 1/4 SDAY. **(G)** Microscopic observation of conidiation of the fungal strains on 1/4 SDAY cultured for 3 d at 26^o^C. Scale bar = 5 μm. *, *P* < 0.05; **, *P* < 0.01; ***, *P* < 0.001.

**
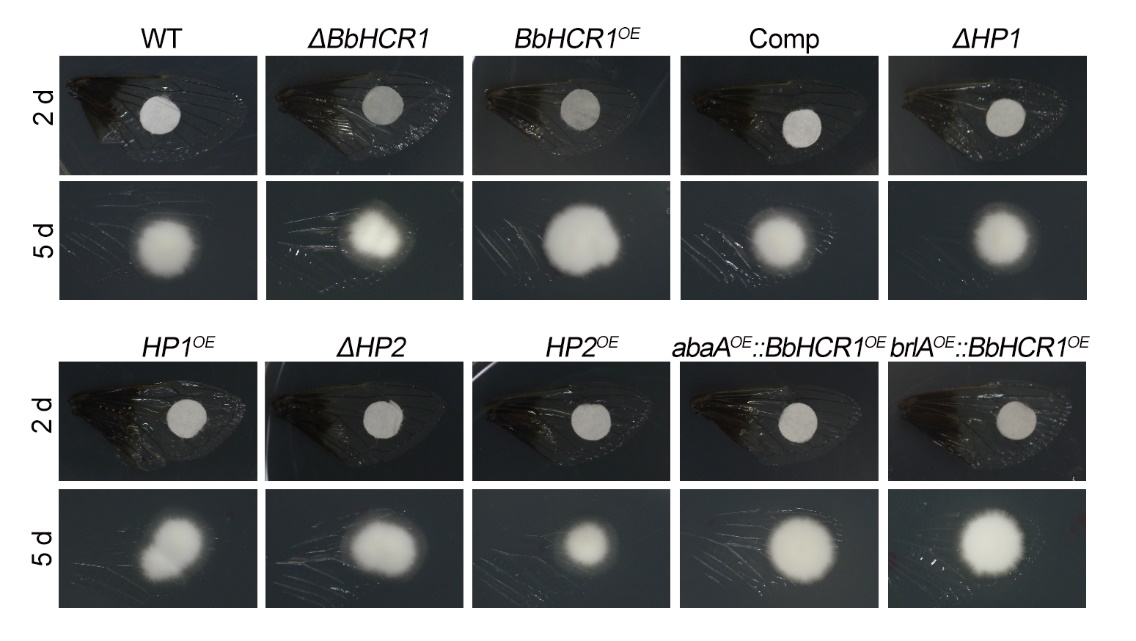
**

**Fig S5.** **Cuticle penetration assays.** Cicada (*Graptopsaltria nigrofuscata*) hind wings were used to mimic the insect cuticle. Two μl of conidial suspension (10^7^ conidia/ml) were spotted onto dissected wings covered on agar plates. Penetrating hyphae were visualized over 3 days.


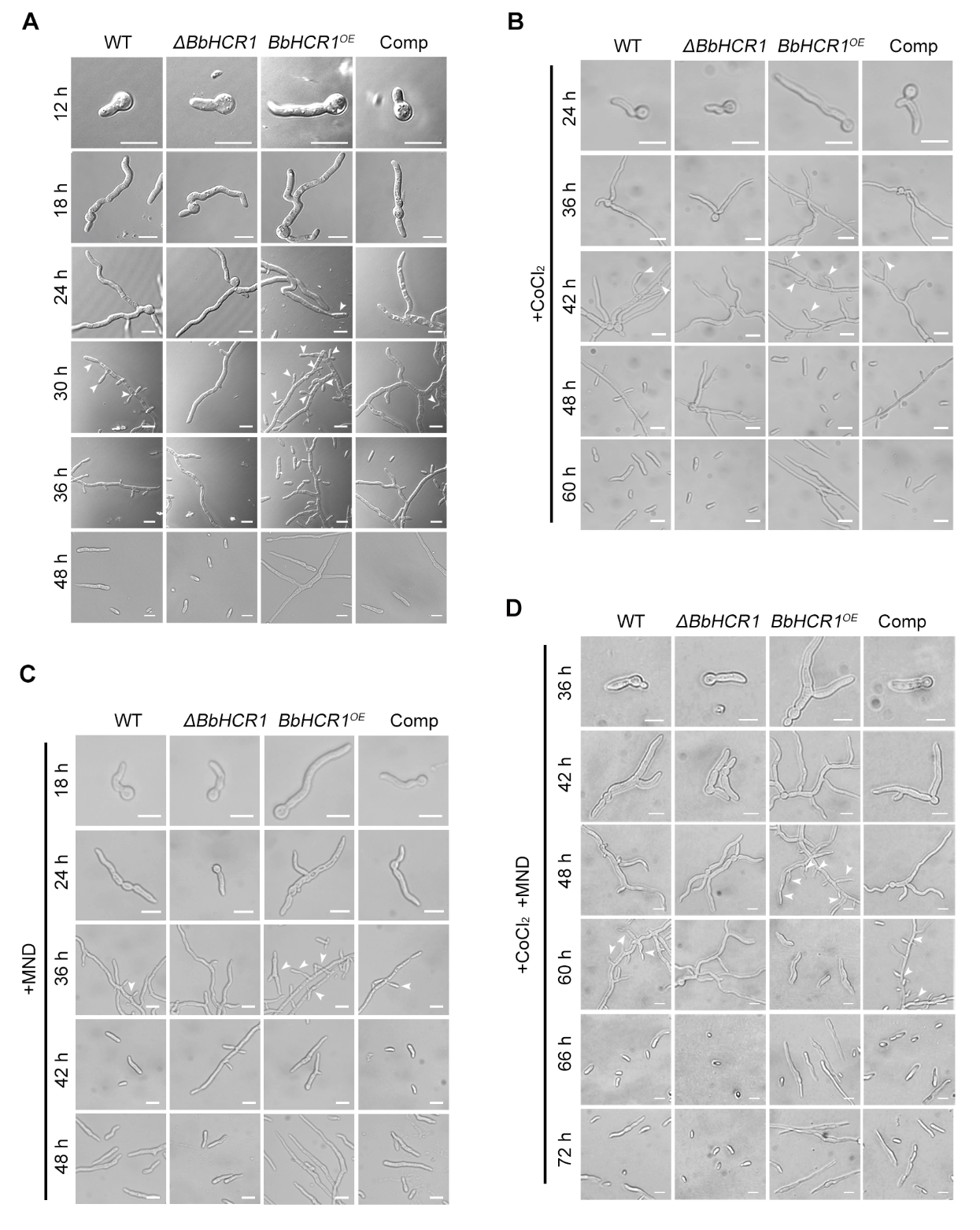


**Fig S6.** **Fungal development and differentiation in broth.** Fungal strains were inoculated by 200 μl of 10^7^ conidia/ml suspension in 20 ml 1/4 SDY **(A)**, 1/4 SDY supplemented with 1 mM CoCl_2_ (+CoCl_2_) **(B)**, 75 mM menadione (+MND) **(C)** or 75 mM MND and 1 mM CoCl_2_ (+CoCl_2_+MND) **(D)** at indicated time. Arrows showed patterns of blastospore formation. Scale bar = 10 μm.

**
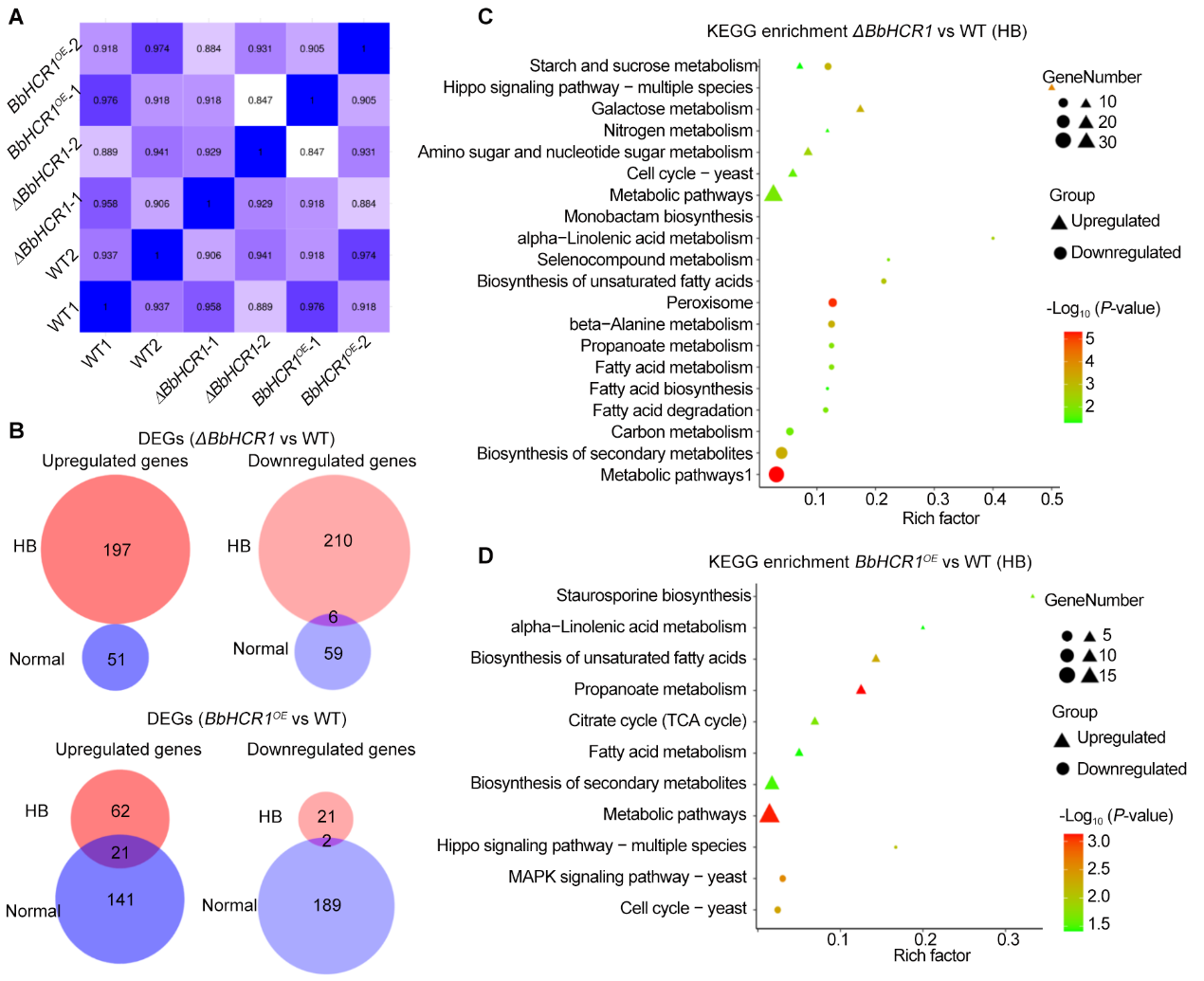
**

**Fig S7.** **RNA-seq analysis from normal cultures and hyphal bodies.** **(A)** Correlation analysis of RNA-seq from two repetitions. **(B)** Venn diagram analysis of *ΔBbHCR1* or *BbHCR1^OE^* versus WT DEGs from normal cultures and hyphal bodies (HB) (|fold change| ≥2, *P*<0.05). **(C-D)** KEGG analysis of *ΔBbHCR1* or *BbHCR1^OE^* versus WT DEGs that specifically expressed in hyphal bodies.


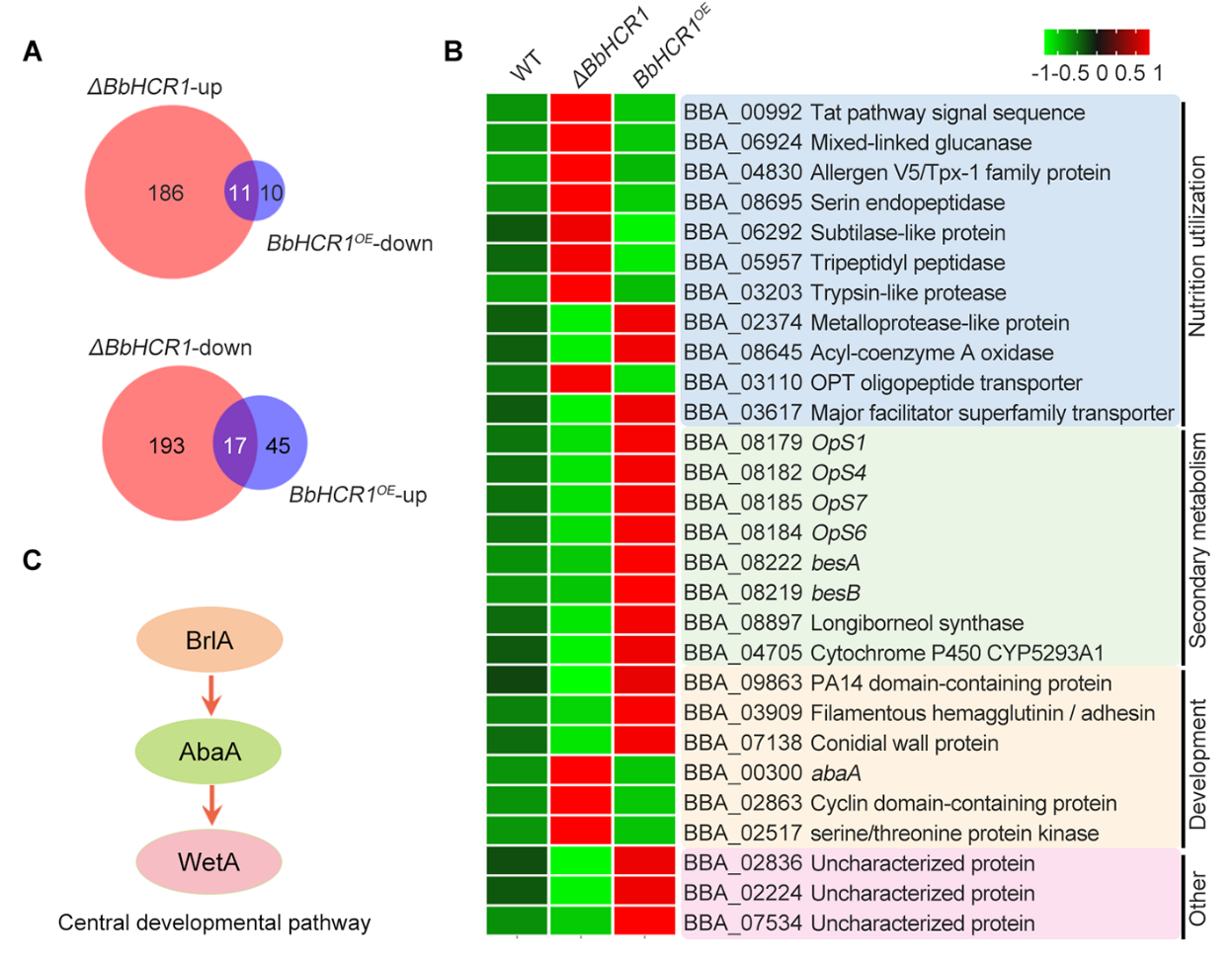


**Fig S8**. **Comparative analysis of *ΔBbHCR1* versus WT and *BbHCR1^OE^* versus WT DEGs special for hyphal bodies.** **(A)** Venn diagram of the DEGs. **(B)** Heat map of 28 DEGs that displayed opposite expression patterns in *ΔBbHCR1* and *BbHCR1^OE^* as compared to the WT. **(C)** Central development pathway. *brlA* and *abaA* genes were upregulated in *ΔBbHCR1* but downregulated in *BbHCR1^OE^* hyphal bodies.


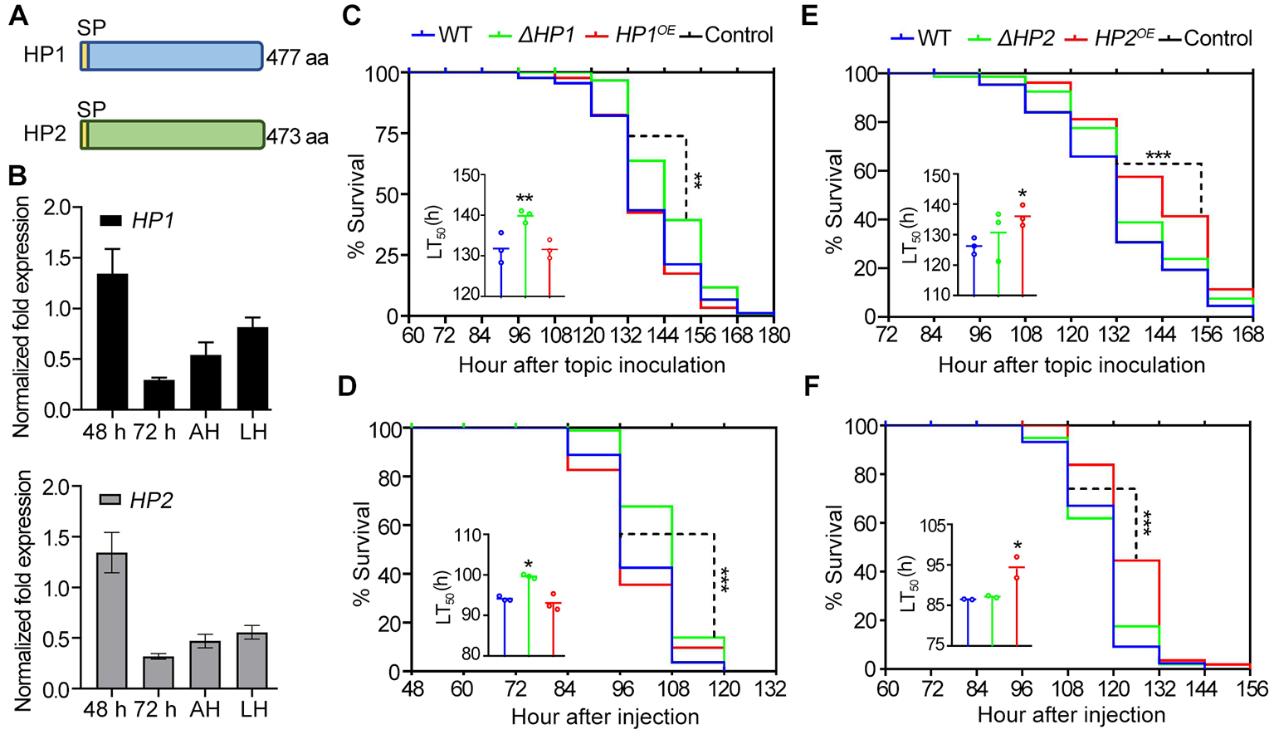


**Fig S9.** **Association of HP1 and HP2 with fungal virulence.** **(A)** Structural diagram of the two proteins. SP indicated the N-terminal signal peptides. **(B)** RT-qPCR analysis of expression patterns from fungal cells proliferated in insect hemocoel after injection of conidia at the indicated hours and in aerial hyphae (AH) and liquid hyphae (LH). **(C-D)** Insect survival and the calculated the LT_50_ values following the topical application (1 ml of 10^7^ conidia/ml) and intrahemocoel injection (2 µl of 10^5^ conidia/ml) of WT, *ΔHP1* and *HP1^OE^* strains. **(E-F)** Insect survival and the calculated the LT_50_ values following the topical application (1 ml 10^7^ conidia/ml) and intrahemocoel injection (2 µl 10^5^ conidia/ml) of WT, *ΔHP2* and *HP2^OE^* strains. *, *P* < 0.05; **, *P* < 0.01; ***, *P* < 0.001.
