# Supplementary material for: A transcription factor-mediated regulatory network controls fungal pathogen colonization of insect body cavities
Source: mBio. 2024 May 15;15(6):e03504-23. doi: 10.1128/mbio.03504-23 (PMC11237471; doi:10.1128/mbio.03504-23)
Supplement: Table S1 — Primers used in this study. [file mbio.03504-23-s0002.docx]

**Table S1 Primers used in this study**

| **Paired primers** | **Sequence (5'–3') *** | **Remarks** |
| --- | --- | --- |
| Real-time RT-PCR analysis of co-expression screening transcription factors genes | | |
| RT1/2  CO1/2  CO3/4  CO5/6  CO7/8  CO9/10  CO11/12  CO13/14  CO15/16  CO17/18  CO19/20 | ACCACAGCCCGTTTATCGTT/ ACGTATGTGAAGCCCGTTGT  CCCATGACACATCACCACCA/ GTCATCCTCGGCCGTAAACT  CGAATACACGACCGAGCTGA/ CAGAATGCGCTGAATCGTGG  GCTATTGGCGCTACCAGACT/ CATCGTGCGCTCTGAAAGTG  TGAGTCAAACTACACGGGGC/ GTAAAAGCCACAGGCCATGC  GTCGAACCTGCTCTCTGTCC/ CTTGAGGATGCTGAGGGTGG  TCACCGACAGACGCCTTTAC/ GTATCGCAAGACGCAGCAAG  GTTACGGGGCCAGCTACAAT/ CTTTTCGCCACCGGTAGACT  CCCGCTACCTGCTACAAGTG/ GATATGTGACCGGCCTCTCC  CAAAGAATGAGAGCGCGCAA/ GCGCAGTTCTTTGGCTTTCA  ATTGACGGCCATTTCGGTCT/ ATCCGATGACATGACCGTGG | *BbHCR1* (BBA_04034)  BBA_03262  BBA_07288  BBA_05123  BBA_03901  BBA_01710  BBA_06675  BBA_08455  BBA_04491  BBA_04109  BBA_04702 |
| Transcription activation | |  |
| A1/2 | CATATGGCCATGGAGGCCGAATTCATGCCTGCGACCGCCGTTG/ CGGCCGCTGCAGGTCGACGGATCCTCAACTTGGAAGCATCACGT | cloning ORF of *BbHCR1* |
| Constructing *GFP* fusion vectors and screening *B. bassiana* transformants | |  |
| C1/2 | aacgacggccagtgccAAGCTTAAGTCTGTGCTGAATCAACAG/ cgcccttgctcaccatGATATCGATGGTGGGCGCGGCAATC | cloning *BbHCR1* promoter |
| C3/4 | aacgacggccagtgccAAGCTTAAGTCTGTGCTGAATCAACAG/ cgcccttgctcaccatGATATCACTTGGAAGCATCACGTTG | cloning *BbHCR1* containing promoter sequence |
| Construction of nucleaus marker vector | | |
| N1/2  N3/4  N5/6  RT1  GFP1 | GTTTAGAGGTAATCCTTCTTtctagaGTTGGGTATGCTCCGGCG/ gatatcTGTTATTGATTAAAAG  CTTTTAATCAATAACAgatatcATGCCTCCCAAGAAGGCTG/ GTTCTCGGAGGAGGCCATgccgccgccTGCCTTGGTGGCGGCCTT  AAGGCCGCCACCAAGGCAggcggcggcATGGCCTCCTCCGAGAAC/ GTAAAACGACGGCCAGTGCCAAGCTTttaggcgccggtggagtgg  ACCACAGCCCGTTTATCGTT  TCTCGTTGGGGTCTTTGCTC | cloning PB3  cloning Histone  cloning RFP  confirmation of transformants with PCR |
| Constructing *BbHCR1* disruption vector p∆BbHCR1 and screening disruption strain | |  |
| L1/2 | ACATGATTACGAATTCGTCAGATTTCTGTGTGCCGC/  CAATATCATCTTCTGTCGAATGAGATGAGGTGCAGGCG | cloning 5'-end of *BbHCR1* |
| B1/2 | CGCCTGCACCTCATCTCATtcgacagaagatgatattg/ TTCTGCAGCTCTTTGCGAGAtcagatctcggtgacgggca | cloning *bar* cassette |
| R1/2 | tgcccgtcaccgagatctgaTCTCGCAAAGAGCTGCAGAA/ CAACACTAGTGGATCCGTCCAAGGGTCGATACGAG | cloning 3'-end of *BbHCR1* |
| S1/2 | CTCGTCCGGTCGCATTCTT/ TTGTGCAGCGATGCTTGGAT | screen or confirmation of disruption strain with PCR |
| Constructing *HP1* disruption vector p∆HP1 and screening disruption strain | |  |
| H1 L1/2 | GCTATGACATGATTACGAATTCTACAGTCACCAAAGCGTAGC/ AATATCATCTTCTGTCGAGTTACGTCCGGTAGAAGCTG | cloning 5'-end of *HP1* |
| H1 B1/2 | CAGCTTCTACCGGACGTAACTCGACAGAAGATGATATT/ CTGCGCCTGTCATATATACGCtcagatctcggtgacggg | cloning *bar* cassette |
| H1 R1/2 | cccgtcaccgagatctgaGCGTATATATGACAGGCGCAG/ CATACCCAACACTAGTGGATCCATAACCGCAAGCGTTAGGTG | cloning 3'-end of *HP1* |
| S3/4 | CCACGTTATCAATATGCCTG/ CATCAAACCATCTAACGGCG | screen or confirmation of disruption strain with PCR |
| Constructing *HP2* disruption vector p∆HP2 and screening disruption strain | |  |
| H2 L1/2 | GCTATGACATGATTACGAATTCGCACAGATTCAAACGTAGGC/ AATATCATCTTCTGTCGACGAGAGAAAAGCAAGCAGAG | cloning 5'-end of *HP2* |
| H2 B1/2 | CTCTGCTTGCTTTTCTCTCGTCGACAGAAGATGATATT/ CGACAATCACCATGGCAAACtcagatctcggtgacggg | cloning *bar* cassette |
| H2 R1/2 | CccgtcaccgagatctgaGTTTGCCATGGTGATTGTCG/ CATACCCAACACTAGTGGATCCGACCAGAAAACGCTAGGTCA | cloning 3'-end of *HP2* |
| S5/6 | GCCGGATTCGGTTTGATTAG/ GATCAATCTGCTGATTCGGC | screen or confirmation of disruption strain with PCR |
| Constructing *abaA* disruption vector p∆abaA and screening disruption strain | |  |
| A L1/2 | GaggtaatccttcttTCTAGAGTCTGGTTGTAATCTCGC/ CAATGTCATCTTCTGTCGACAGCTCAGGCAACAGAGAG | cloning 5'-end of *abaA* |
| Sur 1/2  A R1/2 | GTCGACAGAAGATGACATTG/ GTCGACGTGAGAGCATGCAA  GCATGCTCTCACGTCGACCTGAGCTCGAAGATGCCT/ acgacggccagtgccAAGCTTCATCTTCATGCGCTTCTCC | cloning *sur* cassette  cloning 3'-end of *abaA* |
| S7/8 | AATTTGCAGCTGGCATTGTC/ TCCTCGTTCCTGTCTGCTAA | screen or confirmation of disruption strain with PCR |
| Constructing *brlA* disruption vector and screening disruption strain | |  |
| B L1/2 | GaggtaatccttcttTCTAGATACACGCAACGGAATGAG/ CAATGTCATCTTCTGTCGACGATATACCATCCAGGTGGA | cloning 5'-end of *brlA* |
| Sur 1/2 | GTCGACAGAAGATGACATTG/ GTCGACGTGAGAGCATGCAA | cloning *sur* cassette |
| B R1/2 | GCATGCTCTCACGTCGACCCGACTATGACTTCATGC/ acgacggccagtgccAAGCTTGACAGTCTTGCTCTCTTG | cloning 3'-end of *brlA* |
| S9/10 | ATTCGCCATGAGGCATACTG/ GCACCAAGCAGCAGATGATA | screen or confirmation of disruption strain with PCR |
| Cloning *BbHCR1* sequence used for construction of reverse complement vector pCB-BbHCR1 | |  |
| RC1/2 | gaggtaatccttcttTCTAGACCACTCACTCACCTGCGTG/ acgacggccagtgccAAGCTTAGTGAAGACTTGCAGTGCA | cloning *BbHCR1* containing promoter sequence |
| Cloning *BbHCR1* sequence used for construction of overexpression *BbHCR1* | |  |
| OE 1/2 | CGACCTCGACTCTAGAGGATCCATGAAGCTGGCCTACT/ TATCGAATTCCTGCAGCCCGGGTTATAGCCGAGGCAGGA | cloning *BbHCR1* |
| S11/S12 | TCCATCTCGAGCTTCATTGC/ GTACCATGTGTTTGCTTGCC | confirmation of transformant with PCR |
| Cloning *HP1* sequence used for construction of overexpression *HP1* | |  |
| OE3/4 | TtttaatcaataacaggatccATGAAGGTCGCTTTACTT/ ggtatcgataagcttgatatcTCATGGCACGGCCGATCC | cloning *HP1* |
| Cloning *HP2* sequence used for construction of overexpression *HP2* | |  |
| OE5/6 | TtttaatcaataacaggatccATGCGATACCATAATCTG/ cggtatcgataagcttgatatcTCACAGCAACGCCACACC | cloning *HP2* |
| Cloning *abaA* sequence used for construction of overexpression *abaA* | |  |
| OE7/8 | TtttaatcaataacaggatccATGTCTTCACTATACGCT/ cggtatcgataagcttgatatcCAGTGAATCAAGCACATCA | cloning *abaA* |
| Cloning *brlA* sequence used for construction of overexpression *brlA* | |  |
| OE9/10 | ttttaatcaataacaggatccATGCAGTTTGAGTCGTCG/ cggtatcgataagcttgatatcTCAGTAATCTTCGTGCTTC | cloning *brlA* |
| RT-PCR and real-time RT-PCR analysis of target genes | |  |
| RT3/4  RT5/6  RT7/8  RT9/10 | CTCATCCTGCTAGGCGAGTG/ GTCGATGAGCACCTCTTCCC  TCTTTCCGCGAGAAGACGAC/ CTCCGACAAGACTGTTGGCT  TGATGACTCCCACACACAGC/ TGTCTTGCGCTGAATCTCGT  CAGGACATTCACGTCCCCTT/ GTACATGGCCACCTTGCAGA | *HP1* (BBA_07528)  *HP2* (BBA_01961)  *brlA* (BBA_07544)  *abaA* (BBA_00300) |
| 18S F/R | ACGGGTAACGGAGGGTTAGG/ AGTACACGCGGTGAGGCGGA | *18S rRNA* (BBA_07911) |
| Constructing pET28a vectors | |  |
| PE F/R | CGCGGATCCATGCTGTACTTTACTGACAT/ CCCAAGCTTTTATTGCTTTGTCGGCGTGG | cloning *BbHCR1* cDNA  binding domain |
| \| EMSA \| \| \| \| --- \| --- \| --- \| \| E1/2  E3/4  E5/6  E7/8 \| GAGGTCCTTGTCGACACG/ ACTGGTATGAAGCGTCTG  CGGCCGCAACAAGTGAAC/ GTAACAGATGAAGTGTAC  GGTCGCTCAGCAGCCCGC/ CGCCTCAGCCACGGCGAG  GTCGTACATGATGAACAG/ CAGCTCATTCGCAAAGAG \| *abaA* (BBA_00300)  *HP2* (BBA_01961)  *HP1* (BBA_07528)  *brlA* (BBA_07544) \| | | |
| \| Construction of Y2H vector \| \| \| \| --- \| --- \| --- \| \| AD1/2  BD1/2 \| ATGGCCATGGAGGCCAGTGAATTCATGCCTGCGACCGCCGTTG/  CTGCAGCTCGAGCTCGATGGATCCTCAACTTGGAAGCATCACG  CATATGGCCATGGAGGCCGAATTCATGGCCGAATTTGTTCGC/ CGGCCGCTGCAGGTCGACGGATCCCTATTGTGCTGGAACAGA \| cloning ORF of *BbHCR1*  cloning ORF of *Bbhog1* \| \| BD3/4 \| CATATGGCCATGGAGGCCGAATTCATGTCCCGCGCAAACCCT  CGGCCGCTGCAGGTCGACGGATCCTTACCGCATGATCTCTTGGTA \| cloning ORF of *Bbmpk1* \| | | |
| Phosphorylation site mutant | | |
| P1/2  P3/4  P5/6 | GaggtaatccttcttTCTAGACCACTCACTCACCTGCGTG/ CGACGACGACGTCCTGGGcgcGTGCGACGTATGTGAAGC  CATACGTCGCACgcgCCCAGGACGTCGTCGTCGTC/ cgcCATGGGGCCCATGCTGCC  ATGGGCCCCATGgcgCCGGGGACATCGCAGCGGCCGC/ acgacggccagtgccAAGCTTAGTGAAGACTTGCAGTGCA | S815 to alanine (base:gcg)  T865 to alanine (base:gcg) |
| Constructing Myc fusion vectors and screening *B. bassiana* transformants | |  |
| M1/2  M3/4  M5/6  S13/14 | catgctctcacgtcgacggatccGTTGGGTATGCTCCGGCGCG/ CAACGGCGGTCGCAGGCATtgttattgattaaaagggtg  cacccttttaatcaataacaATGCCTGCGACCGCCGTTG/ ACTTGGAAGCATCACGTTG  CAACGTGATGCTTCCAAGTGAACAAAAGCTAATCTCC/  aaacgacggccagtgccaagcttACAGATCTATATTACCCT CATGCACGCAGTCATATTCG/ TGTTCACCGTCGAGTCCGT | cloning *PB3* sequence  cloning of *BbHCR1*  cloning of 13×Myc and terminator TADH1  confirmation of transformant with PCR |

*Introduced restriction enzyme site are underlined.
